# YAP1 induces invadopodia formation by transcriptionally activating TIAM1 through enhancer in breast cancer

**DOI:** 10.1038/s41388-022-02344-4

**Published:** 2022-06-30

**Authors:** Jie Shen, Qingwen Huang, Weiyi Jia, Shengjie Feng, Liang Liu, Xiaolan Li, Deding Tao, Daxing Xie

**Affiliations:** 1 Molecular Medicine Center, Tongji Hospital, Tongji Medical College, Huazhong University of Science and Technology, Wuhan, PR China; 2 Department of GI Surgery, Tongji Hospital, Tongji Medical College, Huazhong University of Science and Technology, Wuhan, PR China

**Keywords:** Breast cancer, Metastasis

## Abstract

Yes-associated protein 1 (YAP1), a central component of the Hippo pathway, plays an important role in tumor metastasis; however, the underlying mechanism remains to be elucidated. Invadopodia are actin-rich protrusions containing multiple proteases and have been widely reported to promote cell invasiveness by degrading the extracellular matrix. In the present study, we report that YAP1 induces invadopodia formation and promotes tumor metastasis in breast cancer cells. We also identify TIAM1, a guanine nucleotide exchange factor, as a target of the YAP1–TEAD4 complex. Our results demonstrate that YAP1 could promote TEAD4 binding to the enhancer region of TIAM1, which activates TIAM1 expression, subsequently increasing RAC1 activity and inducing invadopodia formation. These findings reveal the functional role of Hippo signaling in the regulation of invadopodia and provide potential molecular targets for preventing tumor metastasis in breast cancer.

## Introduction

Breast cancer is the most common malignant disease in women and causes cancer-related death [1]. Although significant improvements have been achieved in tumor screening and systematic therapy, metastasis remains the main cause of cancer mortality and a major obstacle in breast cancer treatment [2, 3]. Metastasis involves a series of sequential interrelated steps, including detachment from the primary lesion, invasion, extravasation, and the establishment of a microenvironment. Numerous cellular signaling pathways and molecules are involved in these processes [4].

Recently, the Hippo signaling pathway has been reported to be involved in cancer progression and plays an important role in tumor metastasis [5]. The transcriptional coactivator yes-associated protein 1 (YAP1), also known as YAP or YAP65, is the main component of the Hippo pathway. When the Hippo signal is removed, YAP1 is dephosphorylated at serine 127 and enters the nucleus, where it induces target gene expression by binding with related transcription factors, especially TEA domain family members (TEADs) [6, 7]. YAP1 functions as an oncogene, and its hyperactivation leads to various tumor-promoting effects [8–10]. Previous studies have indicated that YAP1 overexpression enhances tumor cell dissemination by promoting intravascular motility and re-entry into the systemic circulation [11]. In addition, YAP1 overexpression can trigger epithelial–mesenchymal transition (EMT) [12, 13] and regulate actin dynamics [14, 15]. These findings demonstrate the critical role of YAP1 in promoting tumor cell metastasis; however, the underlying mechanism remains to be elucidated.

Invadopodia are actin-rich protrusions of the basolateral plasma membrane and are associated with degradation of the extracellular matrix in cancer invasiveness and metastasis [16, 17]. Previous studies have revealed that invadopodia are composed of structural proteins, such as cortactin, TKS4, and TKS5 [18–20], and are regulated by numerous regulatory proteins that control actin dynamics [21]. EMT-related genes, such as TWIST1, and Rho GTPases, such as RAC/CDC42, reportedly play important roles in invadopodia formation [22–25].

In this study, we report that YAP1, which interacts with the transcription factor TEAD4, induces invadopodia formation and promotes tumor metastasis in breast cancer cells. We identified TIAM1, the guanine nucleotide exchange factor, as a target of the YAP1–TEAD4 complex. YAP1 promotes TEAD4 binding to the enhancer region of TIAM1, which upregulates TIAM1 expression, subsequently inducing RAC1 activation and promoting the formation of invadopodia. These findings reveal a novel role of the Hippo signaling pathway in regulating invadopodia formation and provide potential molecular targets for preventing tumor metastasis in breast cancer.

## Results

### YAP1 is necessary and sufficient for invadopodia formation in breast cancer cell lines

**Fig. 1:**
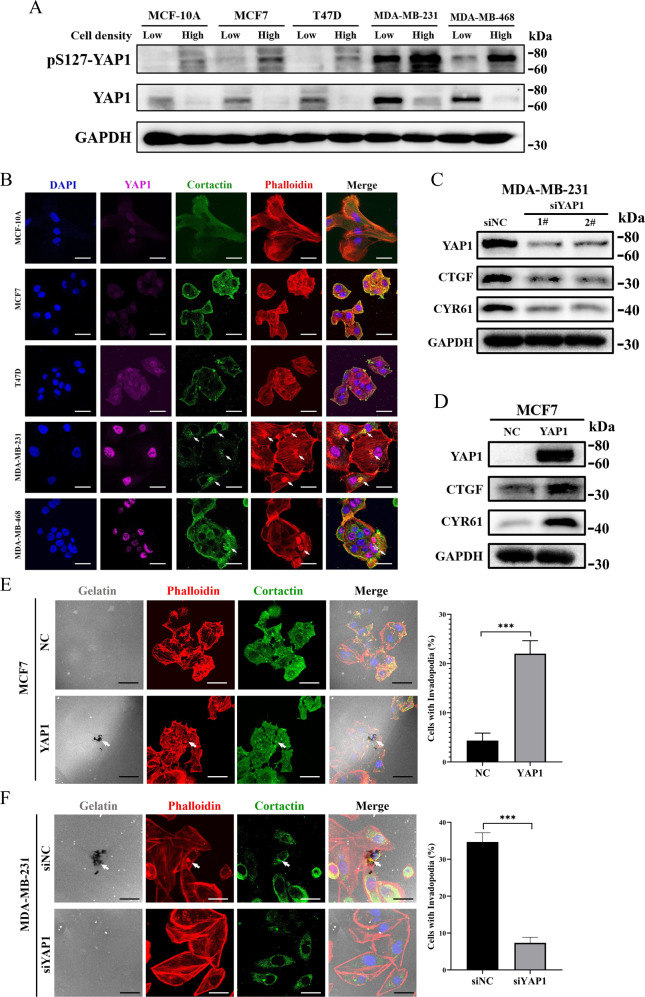
YAP1 is necessary and sufficient for invadopodia formation in breast cancer cell lines. **A** YAP1 protein levels and Ser127 phosphorylation levels (pS127-YAP1) in a non-tumorigenic breast epithelial cell line (MCF-10A) and breast cancer cell lines (MCF7, T47D, MDA-MB-231, MDA-MB-468) with relatively low (1.5 × 10^5^ cells per well in 6-well culture plate) and high cell density (1 × 10^6^ cells per well in 6-well culture plate) were detected using Western blotting. GAPDH was used as a loading control. **B** MCF-10A, MCF7, T47D, MDA-MB-231, and MDA-MB-468 cells were seeded on 0.1% gelatin matrix for 24 h (6 × 10^4^ cells per well in 12-well culture plates), and invadopodia were visualized by colocalization of cortactin (green) and actin (stained by phalloidin, red) (white arrow). YAP1 was marked with anti-YAP1 antibody (magenta). Nuclei were stained with DAPI (blue). Scale bar: 20 µm. **C** Western blot verifying the knockdown of endogenous YAP1 via siRNAs in MDA-MB-231 cells. Cell lysates were probed for YAP1 and its downstream genes (CTGF and CYR61). GAPDH was used as a loading control. **D** Cell lysates from MCF7 control (NC) and MCF7-YAP1 (YAP1) cells were analyzed using Western blot and probed for YAP1, CTGF, CYR61, and GAPDH. **E** MCF7-YAP1 and MCF7-NC cells were seeded on 0.1% Oregon Green™ 488 Conjugate-gelatin matrix (gray) for 24 h (6 × 10^4^ cells per well in 12-well culture plates) and invadopodia were visualized by colocalization of cortactin (green) and F-actin (stained by phalloidin, red) (white arrow). Gelatin degradation appeared as a black area beneath the cells. Nuclei were stained with DAPI (blue). Percentage of cells with invadopodia was quantified. *N* = 100 cells per sample (*n* = 3 per group). ***p < 0.001. Scale bar: 20 µm. **F** MDA-MB-231 cells transfected with scramble siRNA (siNC) and siYAP1-1# (siYAP1) were seeded on 0.1% Oregon Green™ 488 Conjugate-gelatin matrix (gray) for 24 h (6 × 10^4^ cells per well in 12-wells culture plates), and invadopodia were visualized by colocalization of cortactin (green) and F-actin (stained by phalloidin, red) (white arrow). Gelatin degradation appeared as a black area beneath the cells. Nuclei were stained with DAPI (blue). Percentage of cells with invadopodia was quantified. *N* = 100 cells per sample (*n* = 3 per group). ***p < 0.001. Scale bar: 20 µm.

To determine the cellular functions of YAP1 in promoting tumor metastasis and invadopodia formation, we first examined its endogenous expression and Ser127 phosphorylation levels (pS127-YAP1), a marker of YAP1 nuclear-cytoplasmic translocation and inactivation [26], in four breast cancer cell lines (MCF7, T47D, MDA-MB-231, and MDA-MB-468) and a non-tumorigenic breast epithelial cell line (MCF-10A) with relatively low and high cell density. Western blot results showed that YAP1 protein was relatively highly expressed in T47D, MDA-MB-231, and MDA-MB-468 cells, but showed low expression in MCF7 and MCF-10A cells, meanwhile, high cell density could significantly induce YAP1 Ser127 phosphorylation (Fig. 1A). To verify the biological function of YAP1, low cell condition was selected in the subsequent experiments. Next, we examined the presence of invadopodia in the five cell lines with low cell density using immunofluorescence. Invadopodia were scarcely observed in two cancer cell lines (MCF7 and MCF-10A) with YAP1 low expression and T47D cells with YAP1 cytoplasmic retention, and were quite common in MDA-MB-231 and MDA-MB-468 cells with relatively high YAP1 expression and nuclear localization (Fig. 1B). The data indicate that YAP1 is positively associated with invadopodia formation in breast cancer cells.

Next, we tested whether YAP1 is necessary and sufficient for invadopodia formation in breast cancer cells. MCF7 and MDA-MB-231 cells were chosen for further studies, as they exhibit low and high metastatic potential, respectively [27], and invadopodia formation ability. We conducted gain- and loss-of-function experiments by transiently knocking down and stably overexpressing YAP1 in MDA-MB-231 and MCF7 cells, respectively. The protein levels of YAP1 and its downstream targets (CTGF and CYR61) were validated using Western blotting (Fig. 1C, D). Transwell migration, invasion, and wound-healing assays revealed that YAP1 regulates breast cancer cell migration and invasion in vitro (Fig. S1A–D), consistent with our previous results [28]. Immunofluorescence assays showed that YAP1 overexpression induced invadopodia formation and gelatin degradation in MCF7 cells (Fig. 1E), while knockdown of endogenous YAP1 significantly inhibited invadopodia formation and gelatin degradation in MDA-MB-231 cells (Fig. 1F). Furthermore, in 3D tumor spheroid invasion assays, we observed that YAP1 overexpression induced the formation of pseudopod-like structures on the rim of MCF7 cell spheres (Fig. S1E), whereas YAP1 knockdown significantly inhibited MDA-MB-231 cell growth and invasiveness in the 3D culture environment (Fig. S1F). Finally, we also stably overexpressed exogenous YAP1 in MCF-10A cells (Fig. S1G) and verified that YAP1 induces the formation of invadopodia-like structures in non-tumorigenic breast epithelial cells (Fig. S1H). Taken together, these data demonstrated that YAP1 is necessary and sufficient for invadopodia formation in breast cancer cell lines.

### YAP1 induces invadopodia and tumor metastasis in vivo

**Fig. 2:**
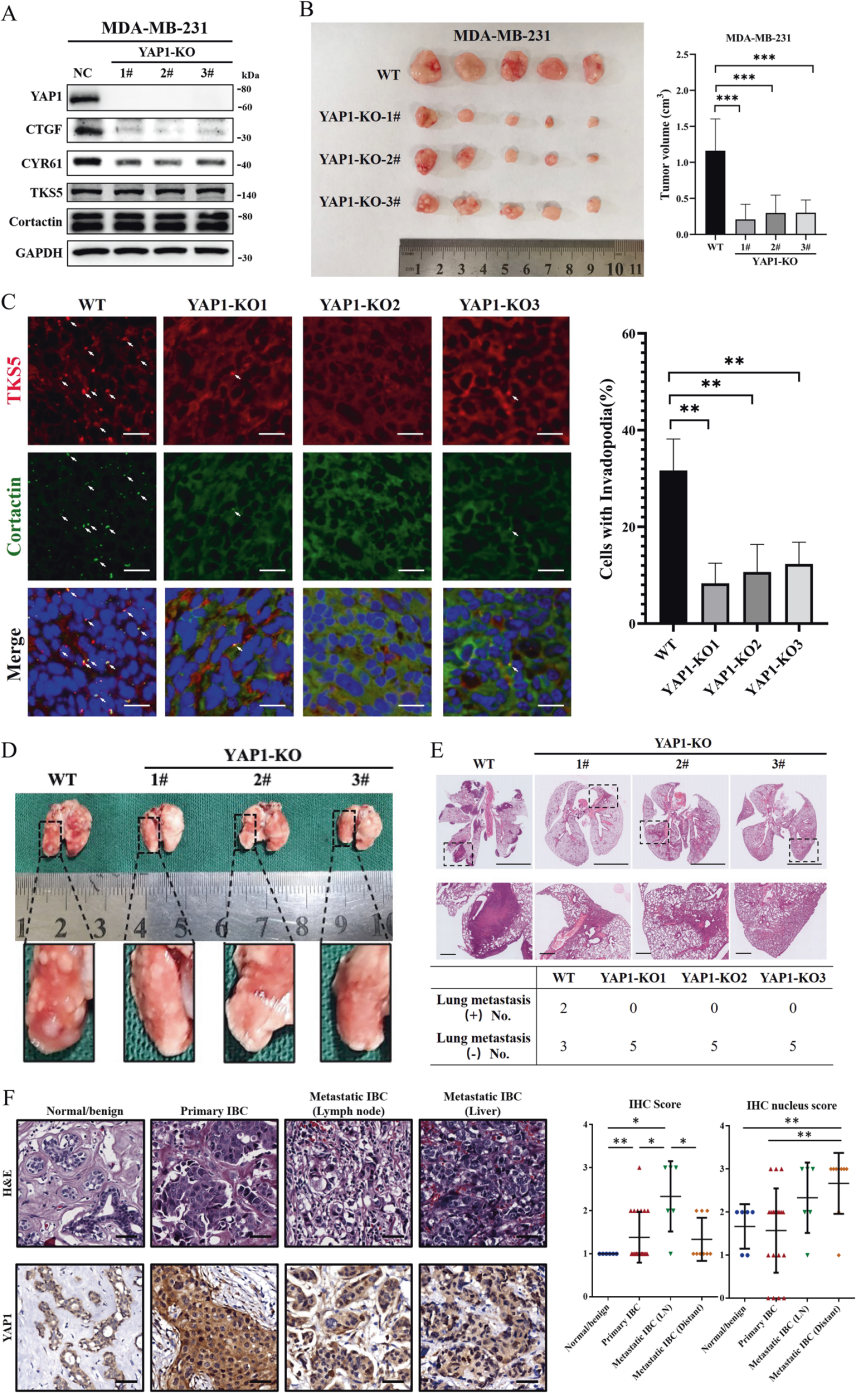
YAP1 induces invadopodia and tumor metastasis in vivo. **A** Cell lysates from MDA-MB-231 control (NC) and YAP1 knockout clones (YAP1-KO1#, 2#, and 3#) cells were analyzed using Western blot and probed for YAP1, CTGF, CYR61, TKS5, cortactin, and GAPDH. **B** Five-week-old female NOD-SCID mice were orthotopically injected with MDA-MB-231 YAP1 knockout cells (YAP1-KOs) or control cells (WT), into gland fat pad. Four weeks after tumor implantation, the xenografted tumors were resected and the tumor volumes were measured. *n* = 5 per group. **C** Images of sections of xenografted tumors stained with TKS5 (red), cortactin (green), and DAPI (blue). Invadopodia were visualized by colocalization of cortactin and TKS5 (white arrow). Percentage of cells with invadopodia was quantified. *N* = 100 cells per sample (*n* = 5 per group). **p < 0.01. Scale bar: 20 µm. **D** Eight weeks after tumor implantation, mice were sacrificed and the lung lobes were collected and examined. **E** H&E staining of the lung lobes showed that lung metastasis occurred in two mice in the WT group, while no nodal metastasis was detected in the YAP1-KOs groups. Scale bar: 5 mm in the upper images and 500 µm in the below images. **F** H&E and YAP1 IHC staining of clinical breast specimens. YAP1 expression level was presented as IHC score, YAP1 nucleus accumulation level was presented as IHC nucleus score. H&E hematoxylin–eosin staining, IHC immunohistochemistry. Normal/benign normal tissue or benign lesion (*n* = 6), primary IBC primary invasive breast cancer specimen (*n* = 21), metastatic IBC (LN) metastatic lymph node of IBC (*n* = 6), metastatic IBC (distant) distant metastatic tumor of IBC (*n* = 9). *p < 0.05; **p < 0.01, Scale bar: 50 µm.

YAP1 has been demonstrated to regulate invadopodia in breast cancer cell lines in vitro. Next, we examined whether YAP1-induced invadopodia formation and promoted tumor metastasis in vivo. We first generated MDA-MB-231 YAP1 knockout (MDA-MB-231-YAP1-KO) and control (MDA-MB-231-NC) cells using the CRISPR-Cas9 technique. To avoid potential clonal effects, three YAP1-KO clones were selected (1#, 2#, and 3#). Western blotting results verified the knockout efficiency of YAP1 and in MDA-MB-231 cells. Notably, YAP1 did not affect the total protein level of invadopodia markers, tyrosine kinase substrate with five SH3 domains (TKS5), and cortactin (Fig. 2A). Moreover, 5-Ethynyl-2’-deoxyuridine staining revealed that all three YAP1-KO clones exhibited significantly lower cell proliferation rates in vitro (Fig. S2A). Transwell migration and Matrigel invasion assays also showed that knocking out YAP1 expression significantly inhibited cell migration and invasiveness in vitro (Fig. S2B). These cell lines were orthotopically injected into the gland fat pad of 5-week-old female NOD-SCID mice. Four weeks after tumor implantation, xenografted tumors were resected and collected. Tumor size measurements showed that YAP1 knockout significantly reduced tumor size (Fig. 2B). Detection of invadopodia by observing colocalization of TKS5, an invadopodia marker in human cancer lines [29], and cortactin in the sections of tumor tissue revealed that knocking out YAP1 expression significantly decreased invadopodia formation (Fig. 2C). Eight weeks after tumor implantation, mice were sacrificed and the lung lobes were examined. Gross observation and hematoxylin–eosin staining showed that lung metastasis occurred in two mice in the WT group, while no nodal metastasis was detected in the YAP1-KO group (Fig. 2D, E). These results indicate that YAP1 promotes invadopodia formation and tumor metastasis in vivo.

To evaluate the expression pattern of YAP1 in patients with invasive breast cancer (IBC), we collected clinical specimens from six normal and benign breast disease tissues, 21 primary tumors, six positive lymph nodes, and nine distant metastatic lesions. IHC assays showed that YAP1 was overexpressed in IBC tissues. Additionally, compared with primary tumors, YAP1 protein levels were significantly increased in positive lymph nodes and distant metastatic lesions, and the YAP1 protein was mainly localized in the nucleus (Fig. 2F, general characteristics of patients are presented in Table S2). Furthermore, gene set enrichment analysis (GSEA) was performed using the GSE database (GSE30480), which contains the expression profile of purified tumor cells from 14 primary breast tumors and six metastatic lymph nodes. GSEA results showed that both the YAP-conserved gene signature and canonical invadopodia regulatory signal, Src [16], were positively associated with lymphatic metastasis (Fig. S2C and Table S3). Prognosis data, which were based on two different datasets, indicated that patients with high YAP1 expression had lower disease-free survival than those with low YAP1 expression (Fig. S2D. GSE21653, *n* = 252, *p* = 0.021 and GSE25006, *n* = 508, *p* < 0.01). To further determine whether YAP1-regulated genes are associated with poor prognosis, we analyzed YAP1 downstream genes, including AMOTL2, CYR61, CTGF, MYC, AREG, GLI2, and AXL [8], in different prognosis groups of IBC patients from The Cancer Genome Talas (TCGA) dataset (*n* = 962) based on the SurExpress program. Our results showed that the expression of YAP1 gene, as well as its target genes (AMOTL2, CYR61, CTGF, MYC, GLI2, and AXL) increased significantly in the poor prognostic group of IBC patients (Fig. S2E, *p* < 0.01). Taken together, these results show that YAP induces invadopodia and tumor metastasis in vivo, and is associated with poor prognosis in patients with IBC.

### YAP1–TEAD4 interaction is essential for invadopodia formation

**Fig. 3:**
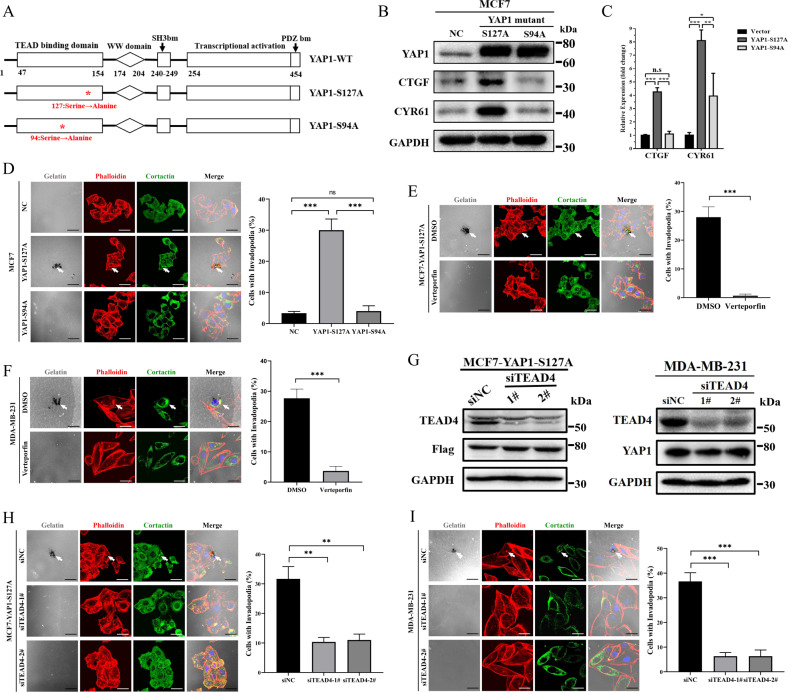
YAP1–TEAD4 interaction is essential for invadopodia formation. **A** Domain organization of YAP1 wild type (YAP1-WT), YAP1 constitutively activated mutant (YAP1-S127A), and YAP1 TEAD-binding domain mutant (YAP1-S94A). Red asterisk indicates the mutation site. **B** Cell lysates from MCF7 control (NC), MCF7-YAP1-S127A, and MCF7-YAP1-S94A cells were analyzed using Western blot and probed for YAP1, CTGF, CYR61, and GAPDH. **C** mRNA expression levels of YAP1 target genes (CTGF and CYR61) in MCF7-NC, MCF7-YAP1-S127A, and MCF7-YAP1-S94A cells. GAPDH was used as an internal control (*n* = 3 per group). *p < 0.05; **p < 0.01; ***p < 0.001 using ANOVA. **D** MCF7-NC, MCF7-YAP1-S127A, and MCF7-YAP1-S94A cells were seeded on Oregon Green™ 488 Conjugate-gelatin matrix (gray) for 24 h (6 × 10^4^ cells per well in 12-well culture plates), and invadopodia were visualized by colocalization of cortactin (green) and F-actin (stained by phalloidin, red) (white arrow). Gelatin degradation appeared as a black area beneath the cells. Percentage of cells with invadopodia was quantified. *N* = 100 cells per sample (*n* = 3 per group). ***p < 0.001. Scale bar: 20 µm. **E** MCF7-YAP1-S127A cells were seeded on 0.1% Oregon Green™ 488 Conjugate-gelatin matrix (gray) and treated with 10 µM verteporfin (DMSO was used as negative control) (6 × 10^4^ cells per well in 12-well culture plates). After culturing for 24 h, invadopodia were visualized by colocalization of cortactin (green) and F-actin (red) (white arrow). Gelatin degradation appeared as a black area beneath the cells. Nuclei were stained with DAPI (blue). Percentage of cells with invadopodia was quantified. *N* = 100 cells per sample (*n* = 3 per group). ***p < 0.001. Scale bar: 20 µm. **F** MDA-MB-231 cells were seeded on 0.1% Oregon Green™ 488 Conjugate-gelatin matrix (gray) and treated with 10 µM verteporfin (DMSO was used as negative control) (6 × 10^4^ cells per well in 12-well culture plates). After culturing for 24 h, invadopodia were visualized by colocalization of cortactin (green) and F-actin (red) (white arrow). Gelatin degradation appeared as a black area beneath the cells. Nuclei were stained with DAPI (blue). Percentage of cells with invadopodia was quantified. *N* = 100 cells per sample (*n* = 3 per group). ***p < 0.001. Scale bar: 20 µm. **G** Western blot verifying knockdown of endogenous TEAD4 via siRNAs in MCF7-YAP1-S127A-Flag and MDA-MB-231 cells. Cell lysates were probed for TEAD4, YAP1, and Flag. GAPDH was used as a loading control. **H** MCF7-YAP1-S127A cells transfected with scramble siRNA (siNC), siTEAD4-1# and siTEAD4-2# were seeded on 0.1% Oregon Green™ 488 Conjugate-gelatin matrix (gray) for 24 h (6 × 10^4^ cells per well in 12-well culture plates), and invadopodia were visualized by colocalization of cortactin (green) and F-actin (stained by phalloidin, red) (white arrow). Gelatin degradation appeared as a black area beneath the cells. Nuclei were stained with DAPI (blue). Percentage of cells with invadopodia was quantified. *N* = 100 cells per sample (*n* = 3 per group). **p < 0.01. Scale bar: 20 µm. **I** MDA-MB-231 cells transfected with scramble siRNA (siNC), siTEAD4-1# and siTEAD4-2#, were seeded on 0.1% Oregon Green™ 488 Conjugate-gelatin matrix (gray) matrix for 24 h (6 × 10^4^ cells per well in 12-wells culture plates), and invadopodia were visualized by colocalization of cortactin (green) and F-actin (stained by phalloidin, red) (white arrow). Gelatin degradation appeared as a black area beneath the cells. Nuclei were stained with DAPI (blue). Percentage of cells with invadopodia was quantified. *N* = 100 cells per sample (*n* = 3 per group). ***p < 0.001. Scale bar: 20 µm.

To determine the functional domain of YAP1 involved in invadopodia regulation, we stably overexpressed two YAP1 mutants (constitutive nuclear-localized (YAP1-S127A-Flag) and TEADs-binding domain mutants (YAP1-S94A-Flag)) in MCF7 cells (Fig. 3A). Compared to YAP1-S94A, the YAP1-S127A mutant accumulated more nuclei (Fig. S3A) and significantly increased the expression of TEADs target genes, such as CTGF and CYR61 (Fig. 3B, C). Using immunofluorescence, we observed that ectopic expression of the YAP1-S127A mutant (MCF7-YAP1-S127A) rather than YAP1-S94A (MCF7-YAP1-S94A) induced invadopodia formation and gelatin degradation in MCF7 cells (Fig. 3D). To further determine the role of the YAP1–TEADs interaction in invadopodia formation, we used verteporfin, a small-molecule inhibitor of the YAP1–TEADs interaction [30]. Immunofluorescence showed that after verteporfin (10 µM) treatment, MCF7-YAP1-S127A cells exhibited decreased invadopodia formation (Fig. 3E). The 3D tumor spheroid invasion assay showed that verteporfin treatment decreased the formation of pseudopod-like structures in MCF7-YAP1 cells (Fig. S3B). Verteporfin also inhibited invadopodia formation and gelatin degradation in MDA-MB-231 cells with relatively high endogenous YAP1 expression (Fig. 3F). In summary, our results suggest a critical role of the interaction between YAP and TEADs family proteins in invadopodia formation.

The TEADs family of proteins includes four highly homologous members (TEAD1, TEAD2, TEAD3, and TEAD4) that exhibit similar biological functions [31, 32]. Public bioinformatics analysis showed that TEAD4, rather than TEAD1-3, was dramatically transcribed among the TEADs family members (Fig. S3C) and was positively correlated with poor prognosis in patients with breast cancer (Fig. S3D). Furthermore, TEAD4 was highly expressed (Fig. S3E) and was able to bind to the exogenous YAP1-S127A mutant in the MCF7 cell line (Fig. S3F). Additionally, endogenous YAP1 was shown to bind to the TEAD4 protein in MDA-MB-231 cells (Fig. S3G). Therefore, we suggest that TEAD4 is a candidate for the YAP1–TEADs complex in our study. Knockdown of endogenous TEAD4 did not affect the protein levels of exogenous or endogenous YAP1 (Fig. 3G); however, it significantly reversed YAP1-induced invadopodia formation in both MCF7-YAP1-S127A and MDA-MB-231 cells (Fig. 3H, I). These results demonstrated that the YAP1–TEAD4 interaction is essential for invadopodia formation in breast cancer cells.

### YAP1–TEAD4 regulates invadopodia through RAC1 activation

**Fig. 4:**
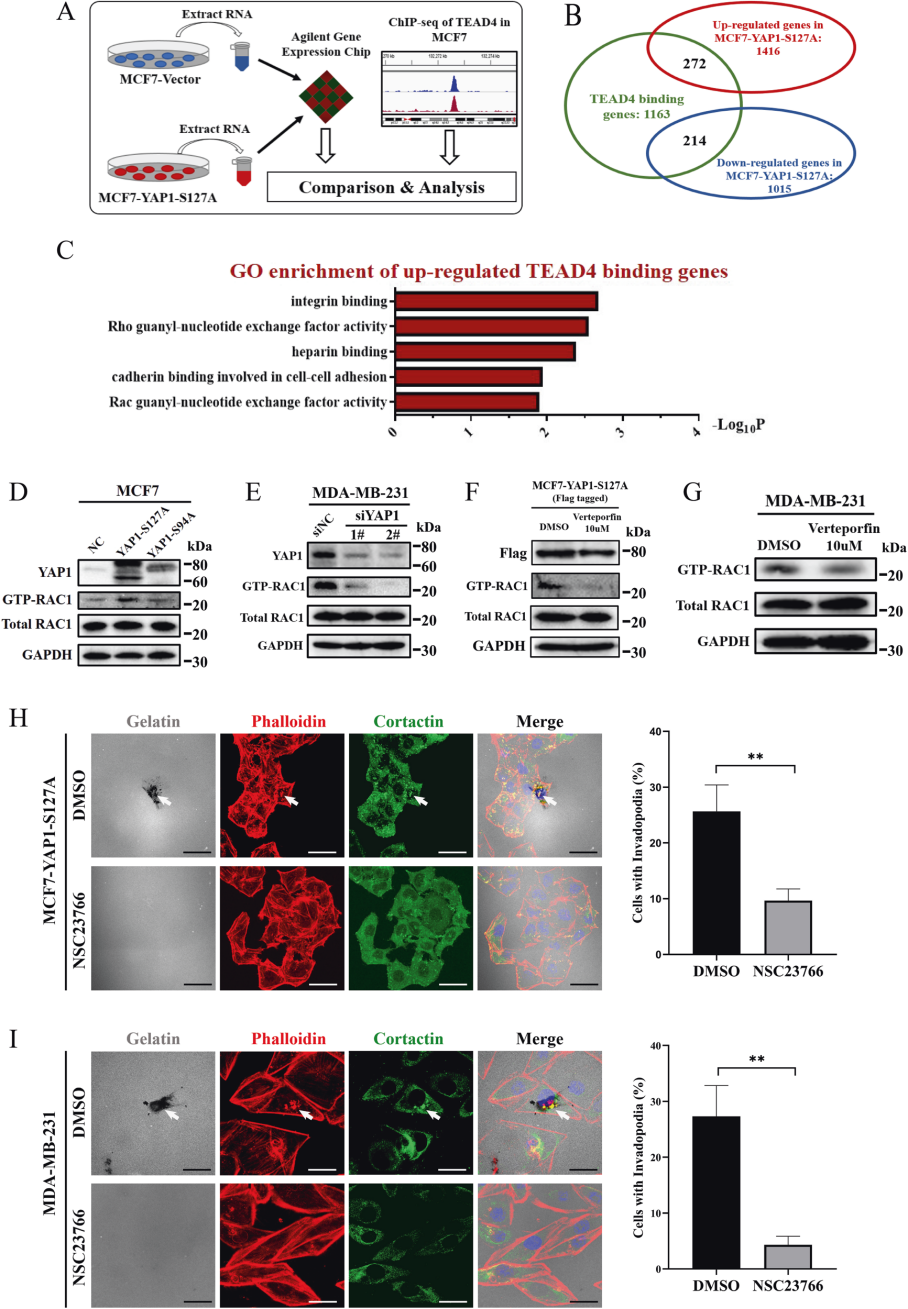
YAP1–TEAD4 regulates invadopodia through RAC1 activation. **A** Experimental design to determine transcriptional targets of the YAP1–TEAD4 complex in MCF7 cells. Total RNAs obtained from MCF7-NC and MCF7-YAP1-S127A cells were subjected to expression profiling using the Agilent SurePrint G3 Human Gene Expression v3 Panel (8 × 60 K, Design ID: 072363). The profiling results were matched with ChIP-seq data of TEAD4 in MCF7 cells from the ENCODE database (GSM1010860). **B** Candidate TEAD4-binding (TB) genes regulated by YAP1-S127A in MCF7 cells. 1163 TB genes acquired from ENCODE were analyzed. Among them, 272 genes were upregulated, and 214 genes were downregulated in MCF7-YAP1-S127A cells. **C** The upregulated TB genes were categorized using GO enrichment analysis. Top five biological processes involved in molecular function are presented. Bar plot representation of Log_10_ *P* values. **D** RAC1 activating form (GTP-RAC1) was obtained from cell lysates of MCF7-NC, MCF7-YAP1-S127A, or MCF7-YAP1-S94A cells via GTP-bound GTPase pull-down assay and analyzed using Western blot. Input lysates were probed for YAP1, total-RAC1, and GAPDH. **E** GTP-RAC1 was obtained from cell lysates of MDA-MB-231 transfected with scramble siRNA (siNC) or siYAP1 (siYAP1-1#, siYAP1-2#) via GTP-bound GTPase pull-down assay and analyzed using Western blot. Input lysates were probed for YAP1, total-RAC1, and GAPDH. **F** After treating with verteporfin or DMSO for 24 h, GTP-RAC1 in cell lysates of MCF7-YAP1-S127A-Flag cells were obtained and analyzed. Input lysates were probed for Flag, total-RAC1, and GAPDH. **G** After treating with verteporfin or DMSO for 24 h, GTP-RAC1 in cell lysates of MDA-MB-231 cells were obtained and analyzed. Input lysates were probed for total-RAC1 and GAPDH. **H** MCF7-YAP1-S127A cells (6 × 10^4^ cells per well in 12-well culture plates) were seeded on 0.1% Oregon Green™ 488 Conjugate-gelatin matrix (gray) and treated with 100 µM NSC23766 (DMSO was used as a negative control). After incubation for 24 h, invadopodia were visualized by colocalization of cortactin (green) and F-actin (red) (white arrow). Gelatin degradation appeared as a black area beneath the cells. Nuclei were stained with DAPI (blue). Percentage of cells with invadopodia was quantified. *N* = 100 cells per sample (*n* = 3 per group). **p < 0.01. Scale bar: 20 µm. **I** MDA-MB-231 cells (6 × 10^4^ cells per well in 12-well culture plates) were seeded on 0.1% Oregon Green™ 488 Conjugate-gelatin matrix (gray) and treated with 100 µM NSC23766 (DMSO was used as a negative control). After incubation for 24 h, invadopodia were visualized by colocalization of cortactin (green) and F-actin (red) (white arrow). Gelatin degradation appeared as a black area beneath the cells. Nuclei were stained with DAPI (blue). Percentage of cells with invadopodia was quantified. *N* = 100 cells per sample (*n* = 3 per group). **p < 0.01. Scale bar: 20 µm.

To identify the functional downstream targets of YAP1–TEAD4, we extracted mRNA from MCF7 cells overexpressing either YAP1-S127A or control plasmid and profiled gene expression using a cDNA microarray (Table S4). These differentially expressed genes (genes with at least a 1.5-fold change in expression after YAP1-S127A induction) were matched with the identified genes containing TEAD4-binding sites in their genomes from the ENCODE ChIP-sequence dataset GSM1010860 (Fig. 4A). Based on this criterion, we screened 272 upregulated TEAD4-binding genes and 214 downregulated TEAD4-binding genes in MCF7 cells (Fig. 4B). GO enrichment analysis was performed to explore the major functions of the YAP1–TEAD4 binding genes. Our analysis implied that “Rho/Rac guanyl-nucleotide exchange factor (GEF) activity” was included in the top five GO enrichment (molecular function) categories of upregulated TEAD4-binding genes (Fig. 4C). These data suggest that the YAP1–TEAD4 complex may regulate Rho/Rac family activity.

Previous studies have reported that RAC1 plays a critical role in invasive protrusion formation and extracellular matrix (ECM degradation [33]. RAC1 activity is required for cortactin tyrosine phosphorylation, a crucial step in invadopodia assembly [34]. GTP binding stimulates the activation of RAC1, and the hydrolysis of GTP into GDP renders it inactive [35, 36]. To examine RAC1 activity, we performed a GST pull-down assay to detect the GTP-bound RAC1. Our data demonstrated that YAP1-S127A mutant significantly increased RAC1 activity in MCF7 cells compared to YAP1-S94A (Fig. 4D). Knockdown of endogenous YAP1 inhibited RAC1 activation in MDA-MB-231 cells (Fig. 4E). Similarly, inhibiting the YAP1–TEAD4 interaction via verteporfin could also reduce RAC1 activity in both MCF7-YAP1-S127A and MDA-MB-231 cells (Fig. 4F, G). CDC42 has also been reported as a small GTPase involved in invadopodia formation [37, 38]. However, overexpression of the YAP1-S127A mutant in MCF7 cells or knockdown/inhibition of endogenous YAP1 in MDA-MB-231 cells could not affect CDC42 activity (Fig. S4A–C). Thus, we conclude that RAC1 may be the key effector of YAP1-induced invadopodia formation in breast cancer cells.

To further determine the functional role of endogenous RAC1 in YAP1-regulated invadopodia, we treated MCF7-YAP1-S127A cells with NSC23766 [39], a RAC1 inhibitor or RAC1-siRNA (Fig. S4D, E). Both treatments blocked YAP1-induced invadopodia formation (Figs. 4H, S4F). Similarly, the inhibition of RAC1 activity reduced invadopodia formation and gelatin degradation in MDA-MB-231 cells (Fig. 4I). Finally, we explored whether CDC42 was involved in YAP1-induced invadopodia formation. However, inhibition of CDC42 activity via ML141 [40] did not significantly reduce invadopodia in MCF7-YAP1-S127A or MDA-MB-231 cells (Fig. S4G, H). These data clearly demonstrated that RAC1 activation is critical for YAP1-regulated invadopodia formation.

### YAP1 mediates RAC1 activation by transcriptionally activating TIAM1 expression

**Fig. 5:**
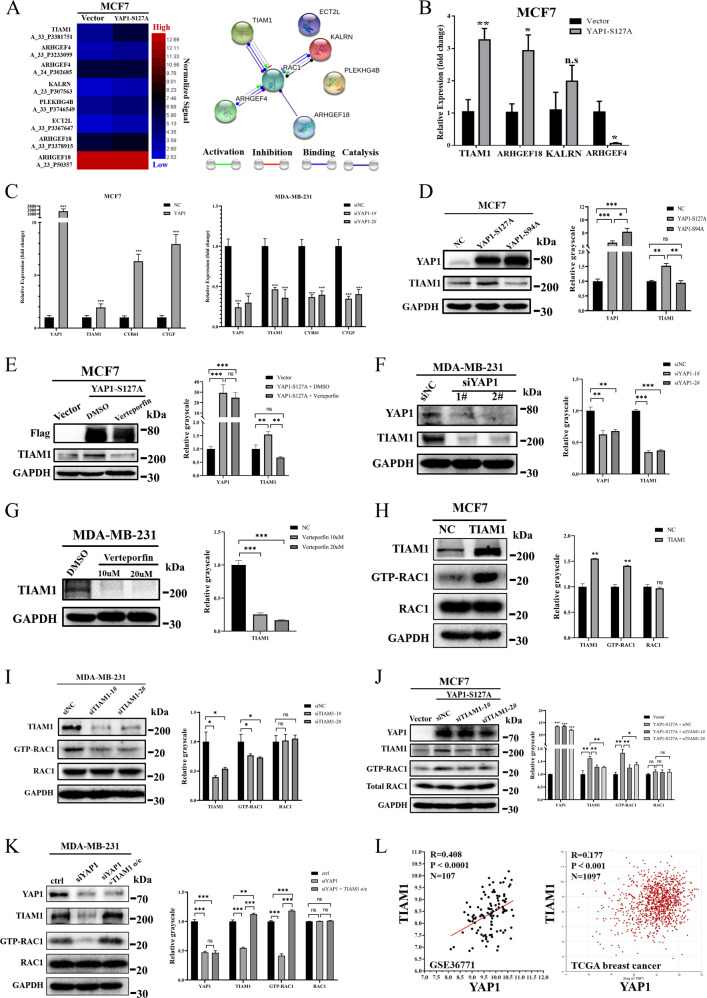
YAP1 mediates RAC1 activation by transcriptionally activating TIAM1 expression. **A** The heatmap presented six TB genes (ECT2L, KALRN, PLEKHG4B, ARHGEF18, ARHGEF4, and TIAM1), which were classified in the Rho/Rac GEF activation GO category (GO: 0005089 and GO: 0030676) and upregulated in MCF7-YAP1-S127A cells. String program was used to identify the interaction between these six genes and RAC1 protein. TIAM1, ARHGEF4, ARHGEF18, and KALRN indicate potential activators of RAC1. **B** RT-qPCR results verified mRNA expression fold-changes of the six candidate genes in MCF7-YAP1-S127A cells compared with MCF7-NC (vector) cells. GAPDH was used as an internal control (*n* = 3 per group). *p < 0.05; **p < 0.01. **C** mRNA expression levels of YAP1, TIAM1, and YAP1 target genes (CTGF and CYR61) in MCF7 cells (transfected with empty vector or YAP1 plasmid) and MDA-MB-231 cells (transfected with siNC, siYAP1-1#, and siYAP1-2# siRNAs). GAPDH was used as an internal control (*n* = 3 per group). ***p < 0.001. **D** Cell lysates from MCF7 control (NC) and MCF7-YAP1-S127A and MCF7-YAP1-S94A cells were analyzed using Western blot and probed for YAP1, TIAM1, and GAPDH. Protein level was quantified via greyscale analysis of the immune blot (*n* = 3). ns non-significant, *p < 0.05; **p < 0.01; ***p < 0.001. **E** Cell lysates from MCF7 control (Vector) and MCF7-YAP1-S127A-Flag cells (treated with DMSO or verteporfin at a dose of 10 µM for 24 h) were analyzed using Western blot and probed for Flag, TIAM1, and GAPDH. Protein level was quantified via greyscale analysis of the immune blot (*n* = 3). ns non-significant, **p < 0.01; ***p < 0.001. **F** Cell lysates from MDA-MB-231 cells (transfected with siNC, siYAP1-1#, and siYAP1-2# siRNAs) were analyzed using Western blot and probed for YAP1, TIAM1, and GAPDH. Protein level was quantified via greyscale analysis of the immune blot (*n* = 3). **p < 0.01; ***p < 0.001. **G** Cell lysates from MDA-MB-231 cells treated with DMSO or verteporfin at a dose of 10 µM for 24 h) were analyzed using western blot and probed for TIAM1 and GAPDH. Protein level was quantified via greyscale analysis of the immune blot (*n* = 3). ***p < 0.001. **H** GTP-RAC1 was obtained from cell lysates of MCF7 cells transfected with empty vector (NC) or TIAM1 plasmid (TIAM1) via GTP-bound GTPase pull-down assay and analyzed using western blot. Input lysates were probed for TIAM1, RAC1 and GAPDH. Protein level was quantified via greyscale analysis of the immune blot (*n* = 3). ns non-significant, **p < 0.01. **I** GTP-RAC1 was obtained from cell lysates of MDA-MB-231 cells transfected with scramble siRNA (siNC) or siTIAM1 (siTIAM1-1#, siTIAM1-2#) via GTP-bound GTPase pull-down assay and analyzed using western blot. Input lysates were probed for TIAM1, RAC1, and GAPDH. Protein level was quantified via greyscale analysis of the immune blot (*n* = 3). ns non-significant, *p < 0.05. **J** GTP-RAC1 was obtained from cell lysates of MCF7-NC cells, and MCF7-YAP1-S127A cells transfected with scramble siRNA (siNC) or siTIAM1 (siTIAM1-1#, siTIAM1-2#) via GTP-bound GTPase pull-down assay and analyzed using Western blot. Input lysates were probed for YAP1, TIAM1, RAC1, and GAPDH. Protein level was quantified via greyscale analysis of the immune blot (*n* = 3). ns non-significant, *p < 0.05; **p < 0.01; ***p < 0.001. **K** MDA-MB-231 was transfected with siYAP1 and overexpressed TIAM1 after knocking down YAP1 expression (siYAP1 + TIAM1 o/e). Scramble siRNA and empty vector were used as control (ctrl). GTP-RAC1 was obtained from cell lysates via GTP-bound GTPase pull-down assay and analyzed using Western blot. Input lysates were probed for YAP1, TIAM1, RAC1, and GAPDH. Protein level was quantified via greyscale analysis of the immune blot (*n* = 3). ns non-significant, **p < 0.01; ***p < 0.001. **L** Gene expression correlation between TIAM1 and YAP1 in clinical breast cancer database. Gene correlation analysis was based on 107 breast cancer patients in Auckland (GSE36771) (left) and 1097 breast cancer patients from TCGA (right). TCGA dataset analysis was based on R2: Genomics Analysis and Visualization Platform.

To further elucidate the mechanism of YAP1-induced RAC1 activation, we focused on six genes with TEAD4-binding sites in their genome (ECT2L, KALRN, PLEKHG4B, ARHGEF18, ARHGEF4, and TIAM1), which were classified in the Rho/Rac GEF activation GO category and upregulated by the YAP1-S127A mutant. We used the String program (string-db.org) to analyze these genes and identified TIAM1, ARHGEF4, ARHGEF18, and KALRN as potential activators of RAC1 (Fig. 5A). RT-qPCR results showed that TIAM1 was the most significantly upregulated gene in MCF7-YAP1-S127A cells (Fig. 5B). Additionally, transient overexpression of exogenous YAP1 and knockdown of endogenous YAP1 significantly upregulated and downregulated the transcriptional level of TIAM1 in MCF7 and MDA-MB-231 cells, respectively (Fig. 5C). Western blotting showed that YAP1-S127A significantly increased TIAM1 protein levels in MCF7 cells, but YAP1-S94A mutant did not (Fig. 5D). Verteporfin also reversed TIAM1 expression in MCF7-YAP1-S127A cells (Fig. 5E). Similarly, knockdown of endogenous YAP1 and inhibition of the YAP1–TEADs interaction reduced TIAM1 protein expression in MDA-MB-231 cells (Fig. 5F, G). Thus, we conclude that TIAM1 is transcriptionally activated by YAP1–TEAD4 signaling.

Next, we investigated whether TIAM1 itself could directly activate RAC1 in both MCF7 and MDA-MB-231 cells using a GST pull-down assay (Fig. 5H, I). To confirm the role of TIAM1 in YAP1–TEAD4 regulated RAC1 activation, we knocked down endogenous TIAM1 in MCF7-YAP1-S127A cells and detected GTP-RAC1. Our results showed that the knockdown of endogenous TIAM1 abolished YAP1-induced RAC1 activation (Fig. 5J). Similarly, TIAM1 overexpression reversed the decrease in GTP-RAC1 caused by YAP1 knockdown in MDA-MB-231 cells (Fig. 5K). We also validated whether the YAP1 paralogous protein, TAZ, had a similar function. Western blot assays showed that knockdown of YAP1 could increase the protein levels of TAZ, and vice versa (Fig. S5A). Additionally, either overexpression or knockdown of TAZ significantly affected transcriptional levels of TIAM1 and RAC1 (Fig. S5B). Considering both proteins could interact with TEAD4 to regulate downstream gene transcription [8], we deduced that the TEAD4 is essential for TIAM1 expression.

Finally, to validate the prevalence of the positive correlation between YAP1 and TIAM1 in clinical specimens, we performed gene correlation analysis based on 107 breast cancer patients in Auckland (GSE36771) and 1097 breast cancer patients from TCGA and found that TIAM1 expression was positively correlated with YAP1 (*n* = 107, *R* = 0.408, *p* < 0.001 in Auckland and *n* = 1097, *R* = 0.177, *p* < 0.001 in TCGA) (Fig. 5L). Analysis of TCGA datasets via the R2 platform also showed that YAP1 and TIAM1 expression exhibited a positive correlation (*R* > 0.1, *p* < 0.01) in almost 30% of all tumor patterns (12 out of 34) (Fig. S5C). Our results indicate that YAP1 mediates RAC1 activation by transcriptionally activating TIAM1.

### TIAM1 is essential for YAP1-induced invadopodia formation

**Fig. 6:**
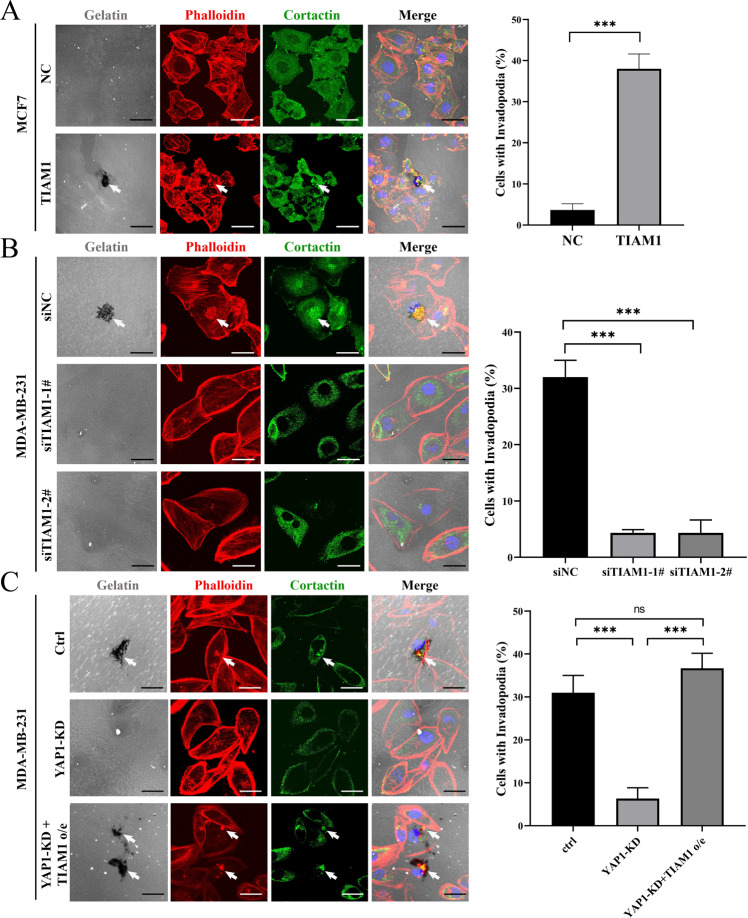
TIAM1 is essential for YAP1-induced invadopodia formation. **A** MCF7 cells transfected with empty vector (NC) or TIAM1 plasmids (TIAM1) were seeded on 0.1% Oregon Green™ 488 Conjugate-gelatin matrix (gray) for 24 h (6 × 10^4^ cells per well in 12-well culture plates), and invadopodia were visualized by colocalization of cortactin (green) and F-actin (stained by phalloidin, red) (white arrow). Gelatin degradation appeared as a black area beneath the cells. Nuclei were stained with DAPI (blue). Percentage of cells with invadopodia was quantified. *N* = 100 cells per sample (*n* = 3 per group). ***p < 0.001. Scale bar: 20 µm. **B** MDA-MB-231 cells transfected with scramble siRNA (siNC), siTIAM1-1# and siTIAM1-2#, were seeded on 0.1% Oregon Green™ 488 Conjugate-gelatin matrix (gray) for 24 h (6 × 10^4^ cells per well in 12-well culture plates), and invadopodia were visualized by colocalization of cortactin (green) and F-actin (stained by phalloidin, red) (white arrow). Gelatin degradation appeared as a black area beneath the cells. Nuclei were stained with DAPI (blue). Percentage of cells with invadopodia was quantified. *N* = 100 cells per sample (*n* = 3 per group). ***p < 0.001. Scale bar: 20 µm. **C** MDA-MB-231 cells with control (ctrl), YAP1 knockdown (YAP1-KD), and YAP1 knockdown plus overexpressing TIAM1 (YAP1-KD + TIAM1 o/e) were seeded on 0.1% Oregon Green™ 488 Conjugate-gelatin matrix (gray) for 24 h (6 × 10^4^ cells per well in 12-well culture plates), and invadopodia were visualized by colocalization of cortactin (green) and F-actin (stained by phalloidin, red) (white arrow). Gelatin degradation appeared as a black area beneath the cells. Nuclei were stained with DAPI (blue). Percentage of cells with invadopodia was quantified. *N* = 100 cells per sample (*n* = 3 per group). **p < 0.01. Scale bar: 20 µm.

Previous data demonstrated that YAP1-regulated invadopodia in a RAC1 dependent manner and identified TIAM1 as a mediator in YAP1-RAC1 signaling. Here, we focus on whether TIAM1 is necessary and sufficient for YAP1-induced invadopodia formation. First, we transiently overexpressed TIAM1 in MCF7 cells and observed enhanced invadopodia formation and gelatin degradation (Fig. 6A). Meanwhile, TIAM1 knockdown significantly decreased invadopodia and gelatin degradation in MDA-MB-231 cells (Fig. 6B).

Next, we transfected TIAM1 overexpression plasmid into YAP1-silenced MDA-MB-231 cells and found that overexpressing TIAM1 could significantly restore invadopodia phenotype (Fig. 6C). In contrast, knocking down endogenous TIAM1 expression in MCF7-YAP1-S127A cells could eliminate YAP1-induced invadopodia formation (Fig. S5D) and gelatin degradation (Fig. S5E). These results indicated that TIAM1 is essential for YAP1-induced invadopodia formation.

### YAP1–TEAD4 transcriptionally activates TIAM1 expression through its enhancer

**Fig. 7:**
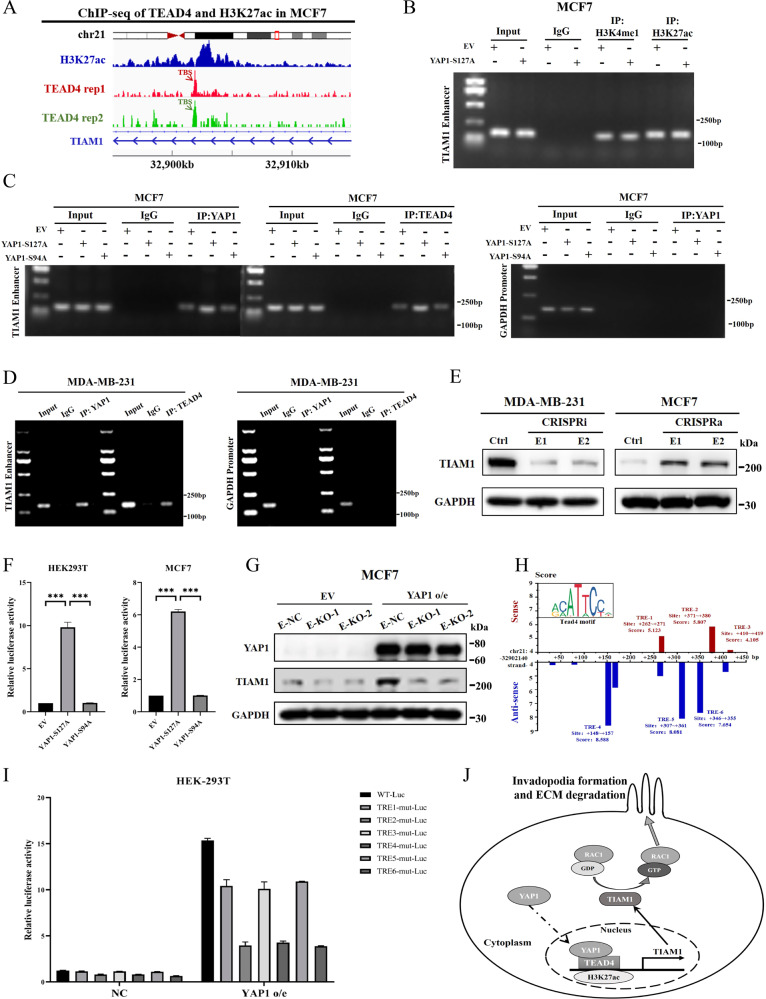
YAP1–TEAD4 transcriptionally activates TIAM1 expression through its enhancer. **A** Diagram of TEAD4 and H3K27ac peak location in the genome region of TIAM1. Analysis was based on ChIP-seq data from ENCODE database (GSM1010860 and GSM945854). TEAD4-binding region was defined as the TEAD4-binding site (TBS). **B** ChIP experiments on TEAD4 and YAP1 were performed using the cell lysates of MCF7-EV (pcDNA3.1) and MCF7-YAP1-S127A cells. Anti-H3K4me1 and anti-H3K27ac antibodies were used for the pull-down experiment. TIAM1 enhancer (TBS of TIAM1) was amplified and measured using agarose gel electrophoresis. Normal IgG was used as a negative control. **C** ChIP experiments on TEAD4 and YAP1 were performed using the cell lysates of MCF7-EV (pcDNA3.1), MCF7-YAP1-S127A and MCF7-YAP1-S94A cells. Anti-TEAD4 and anti-YAP1 antibodies were used for the pull-down experiment. TIAM1 enhancer (TBS of TIAM1) and GAPDH promoter (negative control) was amplified and measured using agarose gel electrophoresis. **D** ChIP experiments on TEAD4 and YAP1 were performed using the cell lysates of MDA-MB-231 cells. TIAM1 enhancer (TBS of TIAM1) and GAPDH promoter (negative control) was amplified and measured using agarose gel electrophoresis. **E** Two sgRNAs targeting TIAM1 enhancer (E1 and E2, ctrl as negative control) were transfected into MDA-MB-231 and MCF7 cells. Then, dCas9-KRAB-MeCP2 plasmid was transfected into MDA-MB-231 for CRISPR-interference (CRISPRi) and dCAS9-VP64 plasmid was transfected into MCF7 for CRISPR-activation (CRISPRa). The protein level of TIAM1 was evaluated via western blot. GAPDH was used as a loading control. **F** HEK293T and MCF7 cells overexpressing control (EV), YAP1-S127A or YAP1-S94A were co-transfected with pGL-Enhancer-TBS plasmid. Then, the transcription activity was evaluated via dual-luciferase reporter assays (*n* = 3 per group). ***p < 0.01. **G** Two sgRNAs (E-KO-1 and E-KO-2) were used to knockout TIAM1 enhancer (TBS of TIAM1) in MCF7-EV and MCF7-YAP1-S127A cells. The protein level of YAP1 and TIAM1 was evaluated via western blot. GAPDH was used as a loading control. **H** JASPAR was used to identify potential TEAD4 regulation elements (TRE) in TBS. Three TREs were in the sense strand (red bar) and eight TREs were in the anti-sense strand (blue bar). All three TREs in the sense strand were marked as TRE1–3, and the three TREs in the anti-sense strand with relatively high JASPAR score (score >7.5) were marked as TRE4–6. **I** HEK293T cells were transfected with control (NC) or YAP1 overexpression plasmid (YAP1 o/e). Dual-luciferase reporter assay was used to identify the transcription activity of TRE1–6 inactivation mutants in pGL-Enhancer-TBS (TRE1–6-mut-Luc). pGL-Enhancer-TBS plasmid (WT-Luc) was used as positive control. *N* = 3 per group. **J** A schematic diagram of the mechanism by which YAP1 regulates TIAM1 expression and induces invadopodia formation.

To characterize the transcriptional regulation of TIAM1 via the YAP1–TEAD4 complex, we annotated the TEAD4 ChIP-sequence data of the MCF7 cell line from the ENCODE database using ChIP-Seek software (http://chipseek.cgu.edu.tw/). Interestingly, we found that the TEAD4-binding site (TBS) was not located in the traditional promoter region of TIAM1 (Table S5). Instead, it was in the region where H3K27ac was enriched, indicating that TBS could be the TIAM1 enhancer region (Figs. 7A, S6A).

To further validate the interaction between YAP1, TEAD4, and TIAM1 enhancer, a ChIP array was performed. First, the active enhancer markers, H3K4me1 and H3K27ac, were demonstrated to interact with TIAM1 enhancer region in MCF7 cells (Fig. 7B). Then, overexpressing YAP1-S127A mutant could induce the binding of TEAD4 and YAP1 toward the enhancer region in MCF7 cells, while the YAP1-S94A mutant appeared to have no such biological effect (Fig. 7C). Furthermore, YAP1 and TEAD4 could interact with the TIAM1 enhancer region in MDA-MB-231 cells (Fig. 7D). Next, we designed two sgRNAs (E1 and E2) to target the region and performed a CRISP inhibition/activation experiment to validate its transcriptional activity. The results showed that inhibiting TIAM1 enhancers with sgRNA in MDA-MB-231 cells significantly reduced TIAM1 protein levels, while activating the enhancer region in MCF7 cells induced TIAM1 expression (Fig. 7E).

To test whether YAP1 regulates the transcriptional activity of the TIAM1 enhancer, we used the pGL3-Enhancer vector to construct a luciferase reporter plasmid containing the TIAM1 enhancer region. The dual-luciferase reporter assay demonstrated that YAP1-S127A, rather than the YAP1-S94A mutant, significantly promoted luciferase activity in both HEK293T and MCF7 cells (Fig. 7F). Additionally, the TIAM1 enhancer region was knocked out using the CRISPR-Cas9 technique (E-KO-1, E-KO-2) (Fig. S6B) and significantly reduced YAP1-induced TIAM1 expression in MCF7 cells (Fig. 7G). These results suggested that the YAP1–TEAD4 complex may activate TIAM1 transcription by binding to its enhancer region.

To identify putative TEAD4-response elements (TREs), we used the JASPAR program to search for the TEAD4 motif (Fig. S6C) in the TIAM1 enhancer region. The results showed that eight potential TREs were in the anti-sense strand of TIAM1 enhancer (blue), and three potential TREs were in the sense strand (Figs. 7H, S6D). To evaluate the activity of each TREs, we first analyzed the frequency matrix profile of TEAD4 from the JASPAR database and found that the sequence “CTGGCAGC” showed low TEAD4-binding ability. Subsequently, we performed a mutation scan to mutate all three TREs in the sense strand (TRE1–3) and the three TREs with relatively high JASPAR score (score > 7.5) in the anti-sense strand (TRE4–6) into “CTGGCAGC”. Dual-luciferase assays showed that mutating these TREs could reverse YAP1 activated luciferase activity in HEK293T cells. Among them, TRE-4 (+148 ~ +157), TRE-6 (+346 ~ +355), and TRE-2 (+371 ~ +380) mutation almost abrogated YAP1-S127A regulated transcriptional activity (Fig. 7I).

In conclusion, our findings reveal a potential mechanism via which YAP1 induces invadopodia formation and promotes tumor cell metastasis in breast cancer (Fig. 7J).

## Discussion

In this study, we demonstrated that YAP1, the key component of Hippo signaling, induces invadopodia formation in breast cancer cells. Furthermore, our findings indicated that YAP1–TEAD4 activated TIAM1 transcription in an enhancer-dependent manner, which subsequently increased RAC1 activity. These findings revealed the critical role of YAP1 in invadopodia formation and provided potential molecular targets for preventing tumor metastasis in breast cancer.

Invadopodia have been widely reported to promote cell invasiveness and metastasis by degrading the extracellular matrix in various cancers [16, 17]. Invadopodia formation is a complex biological process that involves numerous signals, such as Src/Arg kinase, integrin-mediated signaling, EMT-related pathways, Rac GTPase-mediated signaling, and matrix mechano-regulation [16, 21, 33]. RAC1, a member of the Rac-GTPase family, plays a critical role in promoting invadopodia formation [33]. Activation of RAC1 is reportedly required for cortactin tyrosine phosphorylation, which is a crucial step for invadopodia assembly [34]. Here, we demonstrate the important function of YAP1 in invadopodia formation by increasing RAC1 activity. However, considering the reported effects regarding regulating EMT signaling [12] and the presence of the SH3 domain, which exhibits potential catalytic activity for Src kinases [41, 42], we cannot exclude other regulatory mechanisms by which YAP1 regulates invadopodia formation.

Bioinformatics analysis was performed to clarify how YAP1 induces RAC1 activation, and a RAC1-specific guanine nucleotide exchange factor (GEF), TIAM1, was identified as a downstream target of the YAP1–TEAD4 complex. TIAM1 mediates the exchange of guanosine diphosphate (GDP) for guanosine triphosphate (GTP) [43]. The binding of GTP induces a conformational change in RAC1, which results in the activation of RAC1 signaling [35, 36]. Our findings clearly demonstrate that YAP1 upregulates TIAM1 expression and increases RAC1 activation. Intriguingly, a recent study reported that TIAM1 antagonizes the transcription of TAZ/YAP1 target genes by reducing the TAZ/YAP1–TEADs interaction [44]. Thus, negative feedback regulation might exist in the YAP1-TIAM1 axis, which maintains the balance between Hippo signaling and cytoskeleton dynamics.

The molecular mechanism by which YAP1 activates TIAM1 transcription remains unknown. The conventional viewpoint is that YAP1 acts as a transcriptional coactivator via the promoters of downstream genes [45]. However, in breast cancer, genome-wide analysis of YAP1/TAZ-binding sites showed that nearly 91% of YAP1/TAZ-bound cis-regulatory regions coincided with enhancer elements located at a distance from transcription start sites [46]. Our study demonstrated that YAP1–TEAD4 activated TIAM1 transcription by binding to its enhancer region rather than its promoter. Similar effects of YAP1 regulation on enhancers have also been revealed in other studies. Zhu et al. demonstrated that YAP1 functions as a co-regulator of estrogen-regulated genes in breast cancer enhancers [47]. YAP1/TAZ have reportedly flagged a large set of enhancers with super-enhancer-like functional properties [48, 49]. Thus, an enhancer-mediated mechanism may play an important role in YAP1 function, which broadens our understanding of gene regulation in the Hippo pathway [50].

Our study had some limitations. First, we focused on the biological function of YAP1 in invadopodia formation; however, its paralogue, TAZ, has not been considered in the present study. As a homologous protein, TAZ is encoded by paralogous genes of YAP1, and acts as a transcriptional coactivator with 46% amino acid identity [50]. Both proteins interact with the members of the transcriptional enhancer factor, TEADs, to regulate downstream genes transcription and play similar roles in cell biology [50]. Therefore, the role of TAZ in invadopodia should be evaluated in a future study. Second, colocalization of TKS5 and cortactin has been used to detect invadopodia in xenografted tumor. However, the method could not exhibit the dynamic process of invadopodia formation in vivo. Cancer cell invasion events often unpredictably occur deep in complex tissue and are highly dynamic; meanwhile, live-cell fluorescent-based basement membrane reporters are currently not available in vertebrate models [51]. Therefore, detecting and examining the dynamics of invadopodia in native settings is still a challenge [51]. Third, we have unveiled the enhancer region of TIAM1 and demonstrated that YAP1–TEAD4 is essential for TIAM1 transcription. However, enhancers often contain multiple transcription factor binding sites that are required for and control enhancer activity [52]. In addition, the enhancer could remotely regulate multiple genes expression via an enhancer-promoter loop [53]. Therefore, further research on the characteristics and functions of TIAM1 enhancer is required.

In summary, our study demonstrated that YAP1 can upregulate TIAM1 expression by binding to its enhancer, which subsequently activates RAC1 and induces invadopodia formation in breast cancer. These findings reveal the role of Hippo signaling in invadopodia formation and provide potential molecular targets for preventing tumor metastasis in breast cancer.

## Materials/subjects and methods

### Cell culture and transfections

MCF7 and HEK293T cells were cultured in Dulbecco’s modified Eagle medium (DMEM). MDA-MB-468 and MDA-MB-231 cells were cultured in Leibovitz’s L15 medium (L15). The T47D cell line was cultured in RPMI-1640 medium (1640). DMEM, L15, and 1640 culture media were supplemented with 10% fetal bovine serum and 1% penicillin/streptomycin. MCF-10A cells were cultured using the MEGM kit (Cat. #CC-3150, Lonza/Clonetics Corporation, USA). MDA-MB-231 was cultured at 37 °C in a 100% air incubator, and the remaining cells were cultured at 37 °C in a 5% CO_2_ incubator. All the cell lines above were obtained from the American Type Culture Collection. All the cell lines were tested negative for Mycoplasma contamination and authenticated with short tandem repeat assays.

Plasmids pcDNA3.1-YAP1, pcDNA3.1-YAP1-S127A (FLAG-tagged), pcDNA3.1-YAP1-S94A (FLAG-tagged) and pcDNA3.1-TAZ-HA were provided by Prof. Bin Zhao (Zhejiang University). Plasmid pcDNA3.1-TIAM1 (FLAG-tagged) was purchased from GeneChem (Shanghai, China). Small interfering RNAs (siRNAs) used for gene knockdown were provided by Guangzhou RiboBio Co. Ltd. The siRNA sequences are listed in Table S1. Cells were transfected with the indicated plasmids or siRNAs using Lipofectamine 2000 transfection reagent.

### CRISPR/Cas9

YAP1-KO and control MDA-MB-231 cells were engineered using lentivirus (Genechem, Shanghai, China), and after infection and treatment with 2 µg/ml puromycin for 1 week, the remaining cells were trypsinized and seeded into 96-well plates to obtain single-cell clones. Western blotting and Sanger sequencing were used to identify YAP1-KO clones after single-cell clone expansion. sgRNAs targeting YAP1 are listed in Table S1.

CRISPR/Cas9 editing system was also used to delete TIAM1 enhancer in MCF7 cells. Briefly, two sgRNAs were designed using a publicly software, E-CRISP (http://www.e-crisp.org/E-CRISP/) (listed in Table S1). The sgRNAs were then synthetized and cloned into pSpCas9(BB)-2A-Puro (PX459) V2.0 plasmid (Cat.#62988, Addgene, USA) with the BsbI restriction site. The constructed plasmids were co-transfected into MCF7 cells. The MCF7 cells were treated with 1 μg/mL puromycin 36 h after transfection until all the cells of the negative control group died. To obtain single-cell clones, the remaining cells were digested, diluted, and seeded into 96-well plates. Thirty days later, when the cell colonies were visible, the genome DNA were extracted for genotype identification by PCR and Sanger sequencing (primers are listed in Table S1).

### Stable cell lines

Stable YAP1-WT/S127A/S94A overexpressing and control MCF7 cells were generated using a lentivirus system (backbone: pLVX; packaging vectors: psPAX2 and pMD2.G). DNA was amplified from the plasmids and cloned into the pLVX vector. Lentivirus particles were manufactured using HEK293T cells. MCF7 cells were infected and selected using 2 µg/ml puromycin until all cells in the negative control well had died. Western blotting and qPCR were used to detect YAP1 protein and mRNA levels in stable cell lines, respectively.

### Animal experiment

Animal study was performed under the protocols and guidelines approved by the Intitutional Animal Care and Use Comittee of Huazhong University of Science and Technology, Wuhan, China. Briefly, 5-week-old female BALB/c nude mice were purchased from Charles River Inc. (Beijing, China) and were randomly assigned to different groups (*n* = 5 per group). Then, 1 × 10^6^ MDA-MB-231 WT/YAP1-KO (3 clones) cells were trypsinized, collected, and resuspended in 50 μl culture medium, and then injected into the mice mammary fat pads. Four weeks after tumor implantation, the mice were anesthetized and the tumor were removed. Eight weeks after implantation, the mice were sacrificed and the lungs were then harvested for use in further experiments.

Invadopodia in xenografted tumors were examined via immunofluorescence. Briefly, paraffin-embedded tumor slices were routinely dewaxed, rehydrated, and incubated with 1:100 diluted TKS5 (Cat.# A9363, Abclonal, China) /cortactin (Cat.#ab33333, Abcam, UK) antibodies for 2 h at 25 °C after heat-induced epitope retrieval. Cy3- and Alexa Fluor 488 conjugated secondary antibodies were used to visualize TKS5 and cortactin, respectively. The nuclei were stained with 4′-6-diamidino-2-phenylindole (DAPI). Images were obtained using a confocal microscope. Percentage of cells with invadopodia (colocalization of TKS5 and cortactin) was quantified. *N* = 100 cells per sample

### Quantitative real-time PCR (qPCR)

qPCR assay was performed as previously described [54]. The primers used in this research are listed in Table S1. Each assay was repeated thrice.

### Co-immunoprecipitation (Co-IP) assay and western blot assay

Co-IP and Western blot assay were performed as previously described [54], and the antibodies used in the experiment are listed in Table S1.

### Invadopodia assay

Invadopodia assays were performed as previously described [55]. Briefly, 12 mm coverslips were first incubated in 20% nitric acid for 2 h and then washed in ddH_2_O for 4 h. Coverslips were then pretreated with 50 μg/mL poly-L-lysine for 20 min. The coverslips were washed twice with PBS. Next, the coverslips were cross-linked with 0.5% glutaraldehyde for 15 min and washed thrice with PBS. Subsequently, each coverslip was inverted onto a 30 μl droplet of 0.1% Oregon Green 488 Conjugate Gelatin (Cat. #G13186, Life Technologies, USA) for 10 min. The coverslips were then incubated in 5 mg/mL sodium borohydride for 3 min, followed by phosphate-buffered saline (PBS) washes. Coverslips were sterilized with 70% ethanol for 30 min after three PBS washes, placed in a 12-well culture plate, and incubated at 37 °C in complete growth medium for 1 h. Cells (6 × 10^4^ per well) were seeded on each coverslip in 12-well culture plates, cultured for 24 h, and processed for immunofluorescence.

After culturing for 24 h, cells were fixed in 4% paraformaldehyde at 25 °C for 30 min and permeabilized with 0.1% Triton X-100/PBS for 10 min. Nonspecific staining was blocked by incubation with 5% bovine serum albumin/PBS for 2 h. Subsequently, cells were incubated at 4 °C with anti-cortactin antibody at 1:100 dilution overnight, followed by 1:100 IFKine™ Green Donkey Anti-Mouse or Dylight 647 goat anti-mouse secondary antibody (Cat. #A24211; A23610, Abclonal) and 1:100 Alexa 594 phalloidin (Cat. #A22287,Life Technologies) for 2 h. After washing, the nuclei were stained with DAPI, and coverslips were placed on a drop of anti-fading mounting medium on a microscope slide. Images were captured using confocal laser-scanning microscopy at ×600 magnification. Invadopodia were visualized by colocalization of cortactin and F-actin (stained by phalloidin). Gelatin degradation appeared as a black area beneath the cells. Percentage of cells with invadopodia was quantified (*N* = 100 cells per sample). Each experiment was performed in triplicate.

### Cell proliferation assay

Cell proliferation assay was performed via 5-ethynyl-2-deoxyuridine (EdU) Cell Proliferation Kit (Cat.#C10310-1, Ribobio, China) according to the manufactural instruction. Nucleus was stained with Hoechst 33342. Cell proliferation percentage = EdU positive cells/total cells. Each assay was repeated thrice.

### Transwell migration/invasion assay

Transwell migration/invasion assay was performed as previously described [54]. Corning Transwell plates (24 wells, pore size 8 μm, Cat. #3422) were used in the experiment. For the invasion experiments, Transwell filters were coated with 30 μl 1:8 diluted Matrigel (Cat. #356234, BD, USA) on the upper surface of the polycarbonic membrane. Each assay was repeated thrice.

### Wound-healing assay

Cells transfected with the target siRNAs or plasmids were plated in each well of a 6-well culture plate at a cell density of 90%. After cell adhesion, a scratch was created on the following day using a micropipette tip. The migration of cells towards the wound was monitored daily, and images were captured at 24 h time intervals. Each assay was repeated thrice.

### 3D tumor spheroid invasion assay

The method employed for 3D tumor spheroid invasion assays has been described previously [56]. Briefly, stable MCF7-NC/YAP1 cells were diluted to 1 × 10^4^ cells/ml and transferred to ultra-low-attachment 96-well plates (Cat. #7007, Corning, USA) (200 μl per well). Cells were cultured at 37 °C in a 5% CO_2_ incubator for 4 days to obtain tumor spheres. Next, the growth medium was gently removed from the spheroid plates (100 μl/well) and Matrigel was added (100 μl/well; BD, Cat. # 356234). The plates were transferred to a 37 °C incubator for 1 h to allow the Matrigel to solidify, and a complete growth culture medium was added (100 μl/well). After incubation for 72 h, images were captured using an inverted microscope (×200 magnification). Each assay was repeated thrice.

### Microarray gene expression profiling

Total RNA was collected from MCF7-Vector and MCF7-YAP1-S127A cells using TRIzol reagent according to the manufacturer’s protocol. RNA was quantified using a NanoDrop ND-2000 (Thermo Scientific, USA), and RNA integrity was assessed using an Agilent Bioanalyzer 2100 (Agilent Technologies, USA). Sample labeling, microarray hybridization, and washing were performed according to the manufacturers’ standard protocols.

Feature Extraction software (version 10.7.1.1, Agilent Technologies) was used to analyze the array images to obtain raw data. Genespring (version 13.1, Agilent Technologies) was used to complete the basic analysis using the raw data. Differentially expressed genes were identified based on the fold change. The threshold set for upregulated and downregulated genes was a fold change more than 1.5. Gene Ontology (GO) and Kyoto Encyclopedia of Genes and Genomes pathway enrichment of the differentially expressed genes were performed using DAVID software (https://david.ncifcrf.gov/tools.jsp).

### GTP-bound GTPase pull-down assay

GTP-bound GTPase pull-down assays were performed using an Active RAC1/CDC42 Detection Kit (Cat. #8815, 8819, CST, USA). After transfection or verteporfin treatment, MCF7 cells were harvested, according to the manufacturer’s instructions. Enrichment of active GTP-bound GTPases was performed using GST-PAK1-PBD fusion protein beads. Then, the proteins on the beads or total cell lysates were extracted, and Western blotting was performed. The pull-down procedure was performed according to the manufacturer’s instructions.

### Chromatin immunoprecipitation (ChIP)

ChIP was performed using a SimpleChIP Enzymatic Chromatin IP Kit (Cat. #9003, CST) according the manufacturer’s instructions. The antibodies and primers used in the ChIP assay are listed in Table S1.

### Plasmid construction and site-directed mutagenesis

The TEAD4-binding site (TBS) of the TIAM1 genome in MCF7 cells was identified using the ENCODE project (HG19, Chr 21: 32901686-32902140, strand: minus) and synthesized by TsingKe BioTech Co. Ltd. The sequence was cloned into the pGL3-Enhancer vector (Promega, USA) using BglII/HindIII. Site-directed mutagenesis was performed using a Trelief SoSoo Cloning Kit (Cat. #TSV-S1, Tsingke, China), according to the manufacturer’s protocol. The primers are listed in Table S1.

### Dual-luciferase assays

Briefly, 100 ng of pGL3-Enhancer luciferase reporter plasmid with target sequence inserts was co-transfected into HEK293T and MCF7 cell lines with 200 ng of the Control/YAP1-S127A construct and 10 ng of Renilla luciferase pRL-TK plasmid (Promega, Cat. #E2241) using the Lipofectamine 2000 transfection reagent. After 48 h, dual-luciferase assays were performed using the Dual-Luciferase^®^ Reporter Assay System (Promega, Cat. #E1910). Luciferase activity was measured as the ratio of the firefly luciferase signal to the Renilla luciferase signal. All measurements were normalized to those of the control group. Each experiment was performed in triplicate.

### CRISPR activation/interference (CRISPRa/i)

sgRNAs targeting TIAM1 enhancer were designed (listed in Table S1) and cloned into a sgRNA cloning backbone plasmid (Addgene #61424). For CRISPR activation and interfere at the enhancer regions of TIAM1, 2 μg dCAS9-VP64_GFP (Addgene #61422) or dCas9-KRAB-MeCP2 (Addgene #110821) were co-transfected with constructed sgRNA plasmids into MCF7 or MDA-MB-231 cells, respectively. Forty-eight hours after transfection, the cells were harvested for western blot analysis.

### Tissue arrays and immunohistochemistry (IHC)

IHC staining of YAP1 in breast cancer tissue arrays (Shanghai Outdo Biotech Co. Ltd, Cat. #HBre-Duc060CD-01) were performed and scored as previously described [54], using YAP1 antibody (Cat.#14074, CST).

### Public database and bioinformatics analysis

The gene expression profiles of purified tumor cells from 14 primary breast tumor tissues and six metastatic lymph nodes were downloaded from the Gene Expression Omnibus database (GSE30480 [57]) and analyzed using GSEA software (http://software.broadinstitute.org/gsea/) [58]. The clinical data and expression profiles of 252 breast cancer patients (GSE21653 [59]) and 508 breast cancer patients (GSE25066 [60]) were used for Kaplan-Meier analysis using the R2: Genomics Analysis and Visualization Platform (http://r2.amc.nl). Prognosis analysis of The Cancer Genome Atlas (TCGA) breast invasive carcinoma dataset was performed using the SurvExpress program (http://bioinformatica.mty.itesm.mx:8080/Biomatec/SurvivaX.jsp) [61]. The expression level of the TEADs family in breast cancer was determined using the cBioPortal program (www.cbioportal.org) and the Breast Cancer (METABRIC, Nature 2012 & Nat Commun 2016) dataset (samples = 2509) [62, 63]. Gene correlation analysis was performed using R2: Genomics Analysis and Visualization Platform (http://r2.amc.nl) with the Auckland breast cancer dataset (GSE36771 [64]) and TCGA breast cancer datasets. TEAD4 and H3K27ac ChIP-seq data from MCF7 cells were downloaded from the ENCODE project (http://genome.ucsc.edu/ENCODE/downloads.html) (GSM1010860, GSM945854) and visualized using Integrative Genomics Viewer (IGV) software (http://software.broadinstitute.org/software/igv/) [65]. Protein interaction analysis was performed using the STRING database (http://www.string-db.org/) [66]. TEAD4 motif analysis was performed using JASPAR software (http://jaspar.genereg.net/) [67].

### Statistical analysis

Statistical analysis was performed using IBM SPSS (version 19 for Windows; IBM Corp., USA) and GraphPad Prism (version 6.01 for Windows; GraphPad Software Inc., USA). All continuous data are presented as mean ± SD. Continuous data were statistically analyzed using the Student’s *t* test (two-tailed) (comparisons between the two groups) and analysis of variance (comparisons between more than two groups), whereas categorical variables were compared using the *χ*^2^ test and Fisher’s exact test. Statistical significance was set at *p* < 0.05.

## Supplementary information

SUPPLEMENTAL FIGURES AND LEGENDS

Table S1

Table S2

Table S3

Table S4

Table S5

## Data Availability

No statistical methods were used to predetermine sample size. We thus estimated the sample size empirically. No blind method was used in this study. The authors declare that all relevant data of this study are available within the article or from the corresponding author on reasonable request. General characteristics of patients in tissue array are presented in Table S2. The cDNA microarray data can be found in Table S4. Annotation of TEAD4 ChIP-sequence data in MCF7 cell line from ENCODE database (GSM1010860) can be found in Table S5.
