# Supplementary material for: YAP1 induces invadopodia formation by transcriptionally activating TIAM1 through enhancer in breast cancer
Source: Oncogene. 2022 Jun 30;41(31):3830–45. doi: 10.1038/s41388-022-02344-4 (PMC9337990; doi:10.1038/s41388-022-02344-4)
Supplement: Supplementary file 1 — SUPPLEMENTAL FIGURES AND LEGENDS [file 41388_2022_2344_MOESM1_ESM.pdf]

Extended Figures, legends, and tables:

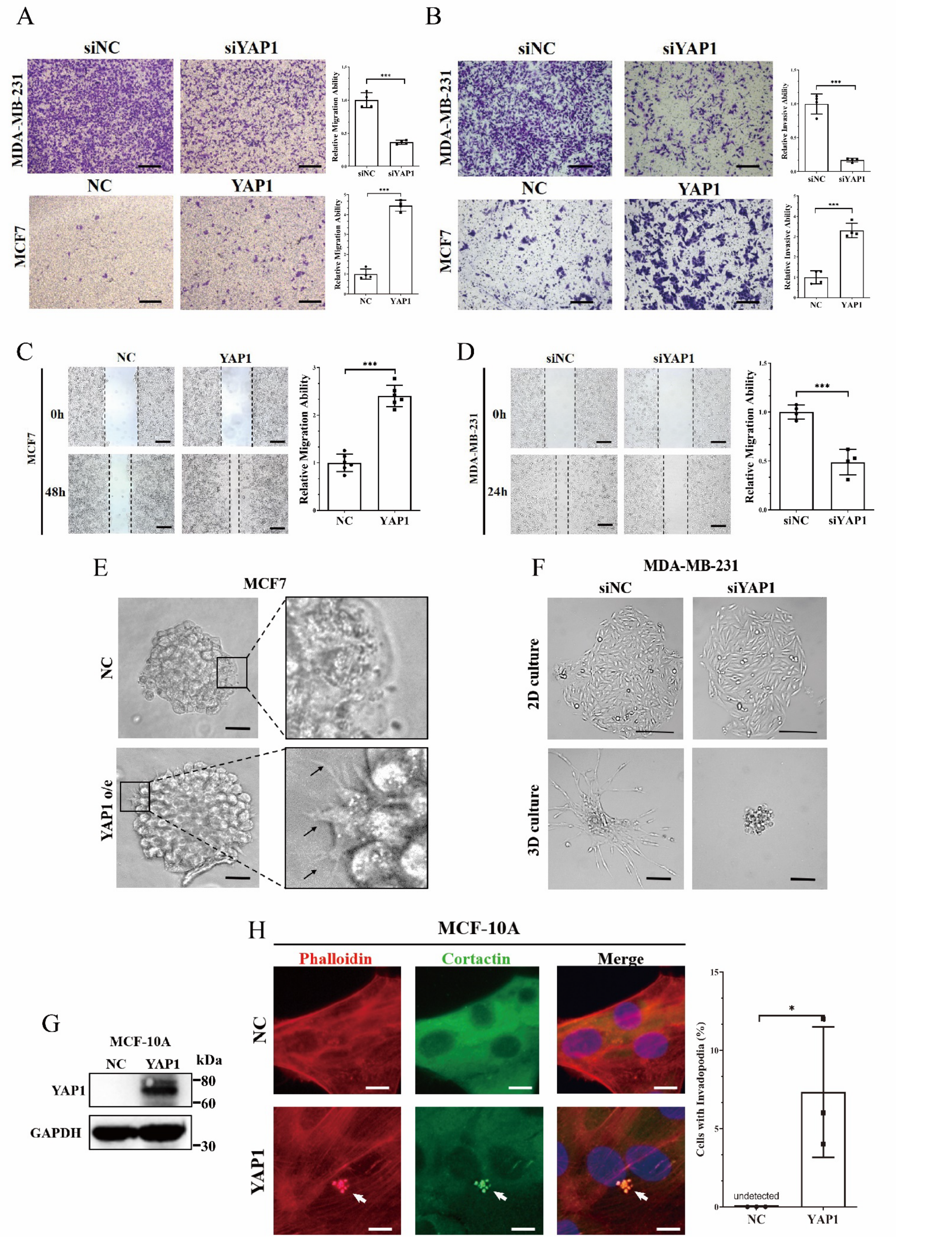

Fig. S1 YAP1 is necessary and sufficient for invadopodia formation in breast cancer cell lines.

(A) MDA-MB-231 (siNC vs. siYAP1) and MCF7 (NC vs. YAP1) cells were used in a Transwell migration

assay. Relative migration ability = migration cells in the test group / migration cells in control group. （n=4 per group）. ***p<0.001. Scale bar: 200 μm.

(B) MDA-MB-231 (siNC vs. siYAP1) and MCF7 (NC vs. YAP1) cells were used in a Transwell invasion assay. Relative invasive ability = invasive cells in the test group / invasive cells in control group. （n=4 per group）. ***p<0.001. Scale bar: 200 μm.

(C) MCF7-NC and MCF7-YAP1 cells with 90% confluency were scratched. Images were taken at 48h time point for the respective control and test groups. Relative migration ability = distance across the wound in test group / distance across the wound in control group. (n=6 per group). ***p<0.001. Scale bar: 200 μm.

(D) MDA-MB-231 cells transfected with siNC or siYAP1 with 90% confluency were scratched. Images were taken at 24h time point for the respective control and test groups. Relative migration ability = distance across the wound in test group / distance across the wound in control group. （n=4 per group）. ***p<0.001. Scale bar: 200 μm.

(E) MCF7-NC and MCF7-YAP1 tumor sphere were cultured in Matrigel 3D culture medium for 3 days and photographed under a phase contrast microscope at 400x. YAP1 overexpression in MCF7 cells promoted pseudopod-like structure (black arrows) formation on the rim of tumor sphere. Scale bar: 40 μm.

(F) MDA-MB-231 cells transfected with siNC or siYAP1 were cultured in traditional 2D culture medium and Matrigel 3D culture medium for 3 days, and photographed under a phase contrast microscope. Scale bar: 200 μm.

(G) Western blot verifying overexpression of YAP1 in MCF-10A cells. Cell lysates were probed for YAP1 and GAPDH.

(H) Stable control (NC) and YAP1 overexpressing (YAP1) MCF-10A cells were seeded on 0.1% gelatin matrix for 24 hours and invadopodia were visualized by colocalization of cortactin (green) and F-actin (stained by phalloidin, red) (white arrow). Nuclei were stained with DAPI (blue). Percent of cells with invadopodia was quantified. N=100 cells per sample（n=3 per group）. *p<0.05. Scale bar: 20 μm.

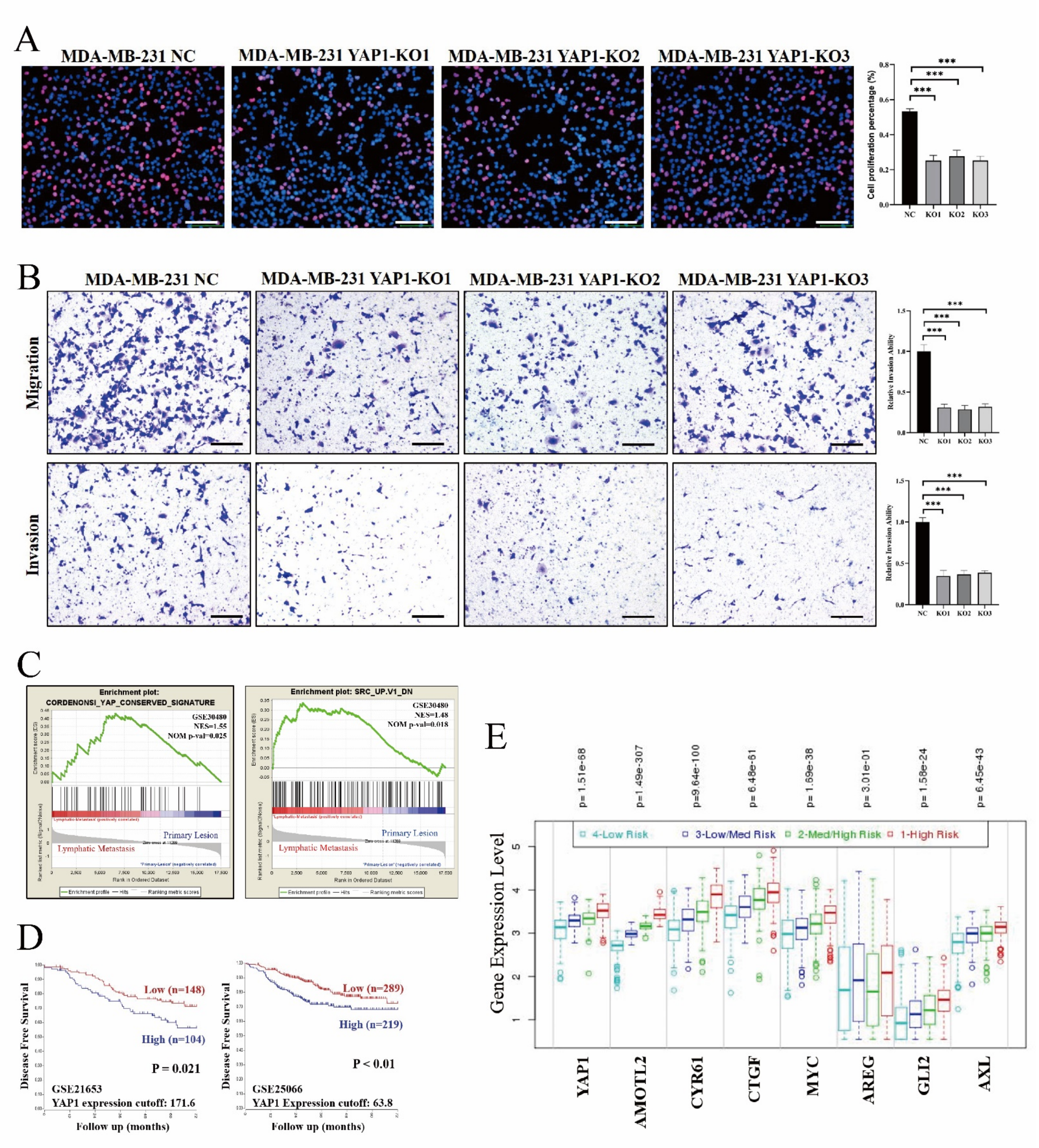

Fig. S2 YAP1 induces cell proliferation, invasiveness, and is associated with poor prognosis in breast cancer. (A) Control (NC) and YAP1 knockout (YAP1-KO1, 2, 3) MDA-MB-231 cells were used in a EdU assay to evaluate the proliferation rate. Cell proliferation percentage = EdU positive cells / total cells. （n=3 per group）. ***p<0.001. Scale bar: 200 μm.

(B) Control (NC) and YAP1 knockout (YAP1-KO1, 2, 3) MDA-MB-231 cells were used in a Transwell migration/invasion assay. Relative migration/invasive ability = migration or invasive cells in the test group / migration or invasive cells in control group. （n=3 per group）. ***p<0.001. Scale bar: 200 μm.

(C) Gene set enrichment analysis (GSEA) of purified tumor cells from 14 primary breast tumor tissues and 6 metastatic lymph nodes from the GEO database (GSE30480). Representative GSEA plots indicated that the conserved YAP1 signature (NES=1.55, NOM p-value=0.025) and Src signal (NES=1.48, NOM p-value=0.018) was positively associated with lymphatic metastasis among 189 oncogenic signature gene sets.

(D) Kaplan-Meier disease-free survival analysis based on YAP1 mRNA expression in 252 breast cancer patients (GSE21653) (Left) and 508 breast cancer patients (GSE25066) (Right). Analysis was based on R2: Genomics Analysis and Visualization Platform.

(E) According to the prognostic condition, IBC patients from TCGA dataset were ranked evenly into four groups (Low, Low-Medium, Medium-High, and High) (left) by risk score. Heat map (middle) and histogram (right) summarizing YAP1 and its target genes (AMOTL2, CYR61, CTGF, MYC, GLI2, AREG and AXL) expression in these IBC patients (ranked by risk score). This prognostic analysis was performed by SurvExpress program.

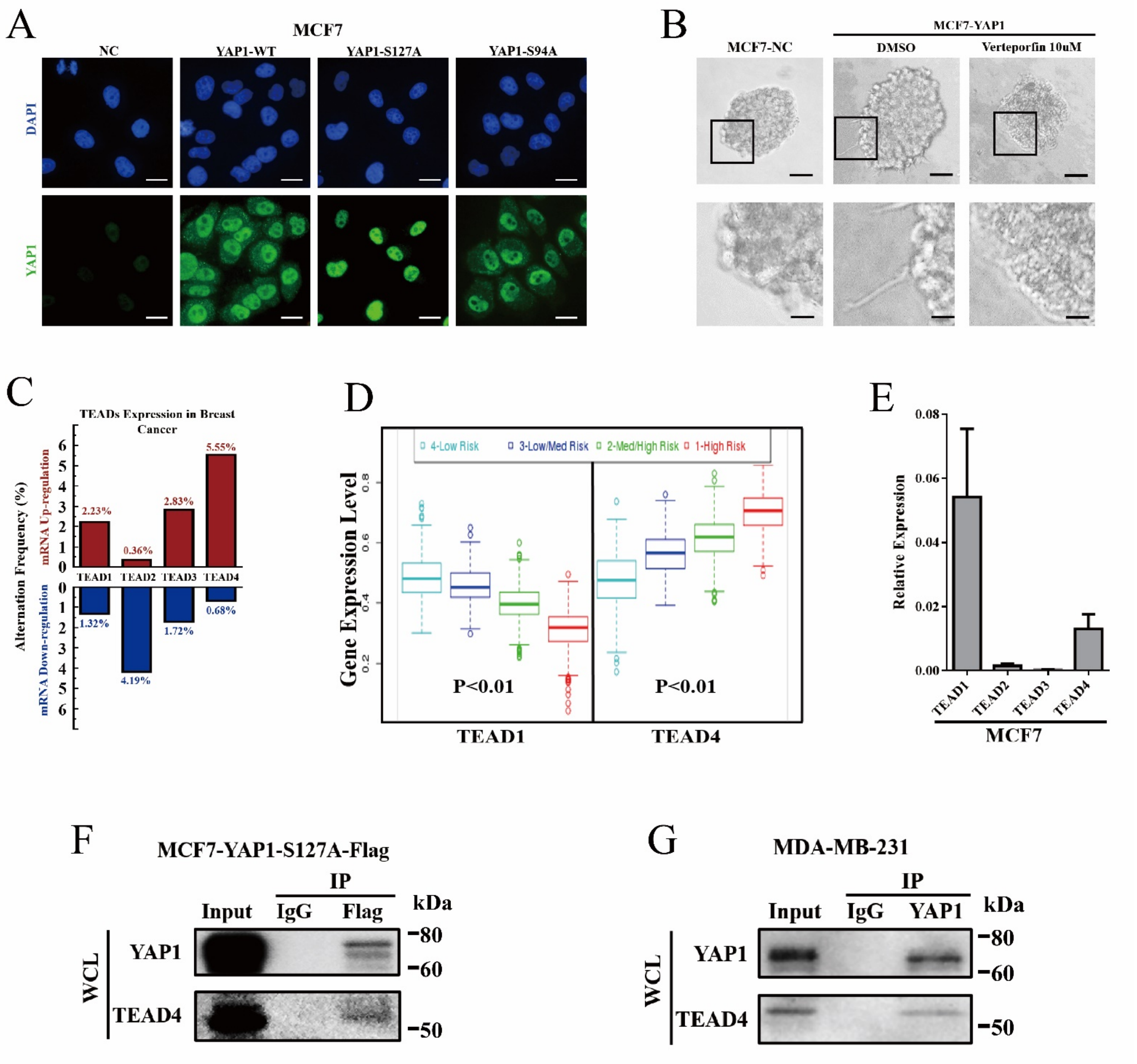

Fig. S3 YAP1-TEAD4 interaction is essential for invadopodia formation.

(A) Subcellular location of YAP1 (green) in MCF7-NC, MCF7-YAP1 (YAP1-WT), MCF7-YAP1-S127A and MCF7-YAP1-S94A was presented via immunofluorescence assay. Nuclei were stained with DAPI (blue). Scale bar: 20 μm.

(B) Tumor spheres of MCF7-NC, MCF7-YAP1 treated with DMSO (as control) and MCF7-YAP1 treated with verteporfin (as test) were cultured in Matrigel 3D culture medium for 3 days and photographed under a phase contrast microscope. Scale bar: 40 μm.

(C) Overview of expression of the TEAD family (TEAD1, TEAD2, TEAD3 and TEAD4) in breast cancer. TEAD4 tended to be the most significantly upregulated among the TEAD proteins. Analysis was based on the cBioPortal program using the Breast Cancer dataset (METABRIC, Nature 2012 & Nat Commun 2016, n=2509).

(D) SurvExpress analysis showed that TEAD4 exhibited a positive correlation with poor prognosis in breast cancer (“Breast cancer recurrence data, 9 datasets from 7 authors” database was used in the analysis).

(E) TEAD1-4 expression levels in MCF7 cells measured by RT-qPCR (n=5 per group). GAPDH was used as an internal control.

(F) Exogenous YAP1-S127A mutant were immunoprecipitated by anti-Flag antibody from MCF7-YAP1-S127A-FLAG cell lysate, analyzed by SDS-PAGE, and probed for YAP1 (Flag) and TEAD4. Normal rabbit IgG was used as negative control. Input lysate was probed for YAP1 and TEAD4.

(G) Endogenous YAP1 were immunoprecipitated by YAP1 antibody from MDA-MB-231 cell lysate, analyzed by SDS-PAGE, and probed for YAP1 and TEAD4. Normal rabbit IgG was used as negative control. Input lysate was probed for YAP1 and TEAD4.

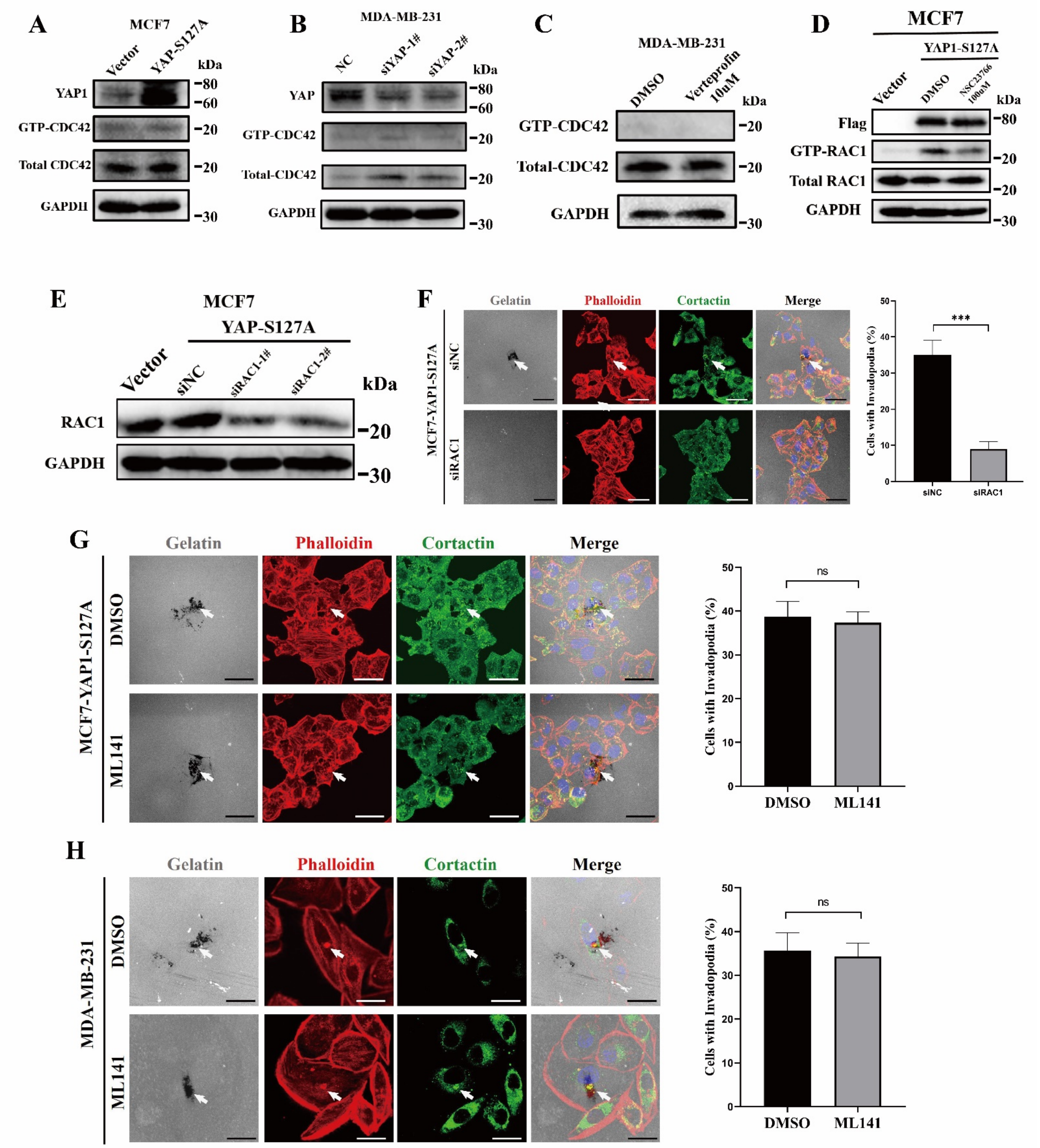

Fig. S4 YAP1-TEAD4 regulates invadopodia formation through RAC1 activation.

(A) CDC42 activating form (GTP-CDC42) was obtained from cell lysates of MCF7-NC and MCF7-YAP1-S127A cells via GTP-bound GTPase pull-down assay and analyzed via western blot. Input lysates were probed for YAP1, total-CDC42 and GAPDH.

(B) GTP-CDC42 was obtained from cell lysates of MDA-MB-231 transfected with scramble siRNA (siNC) or siYAP1 (siYAP1-1#, siYAP1-2#) via GTP-bound GTPase pull-down assay and analyzed via western blot. Input lysates were probed for YAP1, total-CDC42 and GAPDH.

(C) After treating with verteporfin or DMSO for 24 hours, GTP-CDC42 in cell lysates of MDA-MB-231 cells were obtained and analyzed. Input lysates were probed for total-CDC42 and GAPDH.

(D) After treating with verteporfin or NSC23766 for 24 hours, GTP-RAC1 in cell lysates of MCF7-YAP1-S127A-Flag cells were obtained and analyzed. Input lysates were probed for Flag, total-RAC1 and GAPDH.

(E) Western blot verified knockdown of endogenous RAC1 via siRNAs in MCF7-YAP1-S127A cells. Cell lysates were probed for RAC1 and GAPDH.

(F) MCF7-YAP1-S127A cells transfected with scramble siRNA (siNC) and siRAC1-1# (siRAC1) were seeded on 0.1% Oregon Green™ 488 Conjugate-gelatin matrix (grey) for 24 hours and invadopodia were visualized by colocalization of cortactin (green) and F-actin (stained by phalloidin, red) (white arrow). Gelatin degradation appeared as a black area beneath the cells. Nuclei were stained with DAPI (blue). Percent of cells with invadopodia was quantified. N=100 cells per sample（n=3 per group）. $^{**}p<0.01$. Scale bar: 20 μm.

(G) MCF7-YAP1-S127A cells were seeded on 0.1% Oregon Green™ 488 Conjugate-gelatin matrix (grey) and treated with 100 μM ML141 (DMSO was used as a negative control). After 24h of culture, invadopodia were visualized by colocalization of cortactin (green) and F-actin (red) (white arrow). Gelatin degradation appeared as a black area beneath the cells. Nucleus was strained with DAPI (blue). Percent of cells with invadopodia was quantified. N=100 cells per sample（n=3 per group）. Scale bar: 20 μm.

(H) MDA-MB-231 cells were seeded on 0.1% Oregon Green™ 488 Conjugate-gelatin matrix (grey) and treated with 100 μM ML141 (DMSO was used as a negative control). After 24h of culture, invadopodia were visualized by colocalization of cortactin (green) and F-actin (red) (white arrow). Gelatin degradation appeared as a black area beneath the cells. Nuclei were stained with DAPI (blue). Percent of cells with invadopodia was quantified. N=100 cells per sample（n=3 per group）. Scale bar: 20 μm.

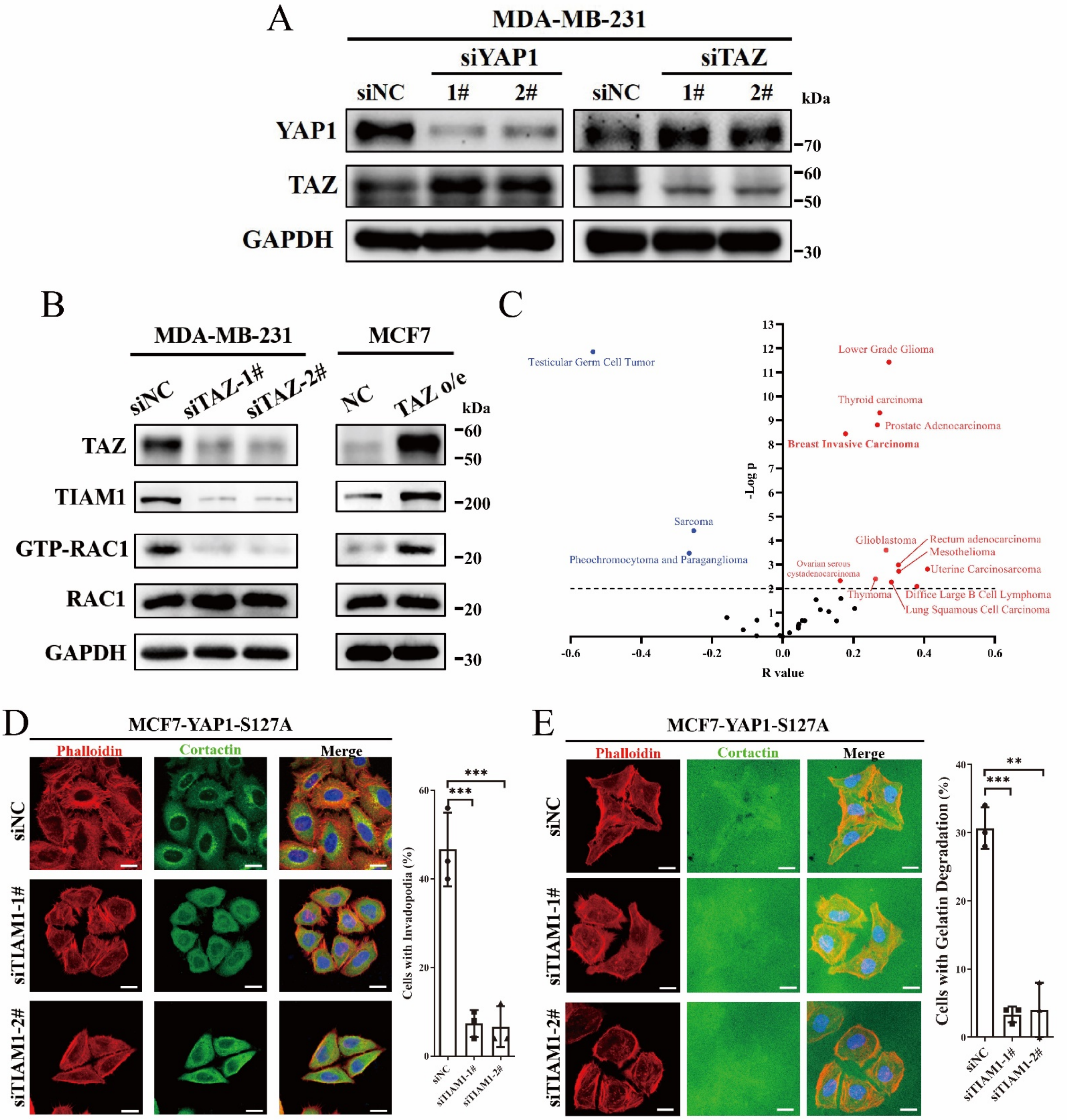

Fig. S5 YAP1/TAZ regulates TIAM1 and RAC1 activity.

(A) Cell lysates from MDA-MB-231 control (siNC), YAP1 knockdown (siYAP1-1#, siYAP1-2#) and TAZ knockdown (siTAZ-1#, siTAZ-2#) cells were analyzed using western blot and probed for YAP1, TAZ and GAPDH.

(B) GTP-RAC1 was obtained from cell lysates of MDA-MB-231 transfected with scramble siRNA (siNC) or siTAZ (siTAZ-1#, siTAZ-2#) and MCF7 with control (NC) or TAZ overexpressing (TAZ o/e) via GTP-bound GTPase pull-down assay and analyzed via western blot. Input lysates were probed for TAZ, TIAM1, total-RAC1 and GAPDH.

(C) Gene expression correlation between TIAM1 and YAP1 in multiple tumor patterns in TCGA database. Across TCGA datasets, analysis was based on R2: Genomics Analysis and Visualization Platform.

(D) MCF7-YAP1-S127A cells transfected with scramble siRNA (siNC) and siTIAM1 (1# and 2#) were seeded on 0.1% gelatin matrix for 24 h, and invadopodia were visualized by colocalization of cortactin (green) and F-actin (stained by phalloidin, red). Nuclei were stained with DAPI (blue). Percentage of cells with invadopodia was quantified. N=100 cells per sample (n=3 per group). ***$p<0.001$. Scale bar: 20 μm.

(E) MCF7-YAP1-S127A cells transfected with scramble siRNA (siNC) and siTIAM1 (1# and 2#) were plated on Alexa Fluor 488-conjugated gelatin (green) for 24 h. F-actin was stained with phalloidin (red), and nuclei were stained with DAPI (blue). Gelatin degradation appeared as a black area beneath the cells. Percentage of cells with gelatin degradation was quantified. N=100 cells per sample (n=3 per group). **$p<0.01$, ***$p<0.001$. Scale bar: 20 μm.

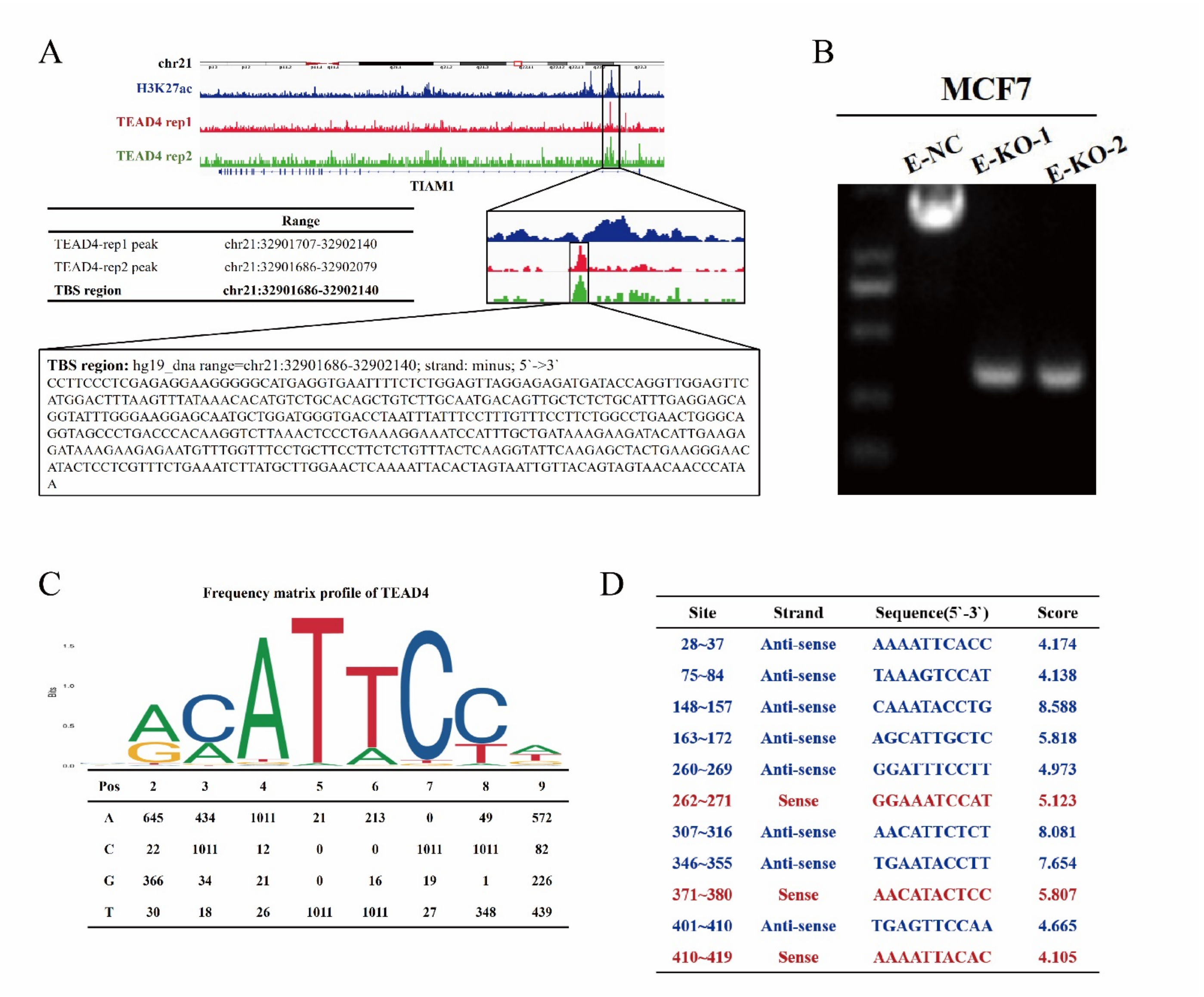

Pos	2	3	4	5	6	7	8	9
A	645	434	1011	21	213	0	49	572
C	22	1011	12	0	0	1011	1011	82
G	366	34	21	0	16	19	1	226
T	30	18	26	1011	1011	27	348	439

Site	Strand	Sequence(5`-3`)	Score
28~37	Anti-sense	AAAATTCACC	4.174
75~84	Anti-sense	TAAAGTCCAT	4.138
148~157	Anti-sense	CAAATACCTG	8.588
163~172	Anti-sense	AGCATTGCTC	5.818
260~269	Anti-sense	GGATTTCCTT	4.973
262~271	Sense	GGAAATCCAT	5.123
307~316	Anti-sense	AACATTCTCT	8.081
346~355	Anti-sense	TGAATACCTT	7.654
371~380	Sense	AACATACTCC	5.807
401~410	Anti-sense	TGAGTTCCAA	4.665
410~419	Sense	AAAATTACAC	4.105

Fig. S6 YAP1-TEAD4 transcriptionally activates TIAM1 expression through its enhancer.

(A) IGV analysis of ChIP-seq data from the ENCODE database (GSM1010860 and GSM945854). The TBS region is calculated as the aggregate of the TEAD4 binding peaks from two bio-replicates of ChIP-seq experiments. The TBS sequence is presented in the figure.

(B) Two sgRNAs (E-KO-1 and E-KO-2) was used to delete TIAM1 enhancer in MCF7 cells via CRISPR/Cas9. PCR and agarose gel electrophoresis were used to verified the knock out efficiency.

(C) Frequency matrix profile of TEAD4.

(D) Detailed information of potential TEAD4 regulation elements (TRE) in TBS.

Table S1 (separate file). Reagents and materials.

Table S2 (separate file). General characteristics of clinical specimens.

Table S3 (separate file). Gene set enrichment analysis of C6_Oncogenic signatures in GSE30480 dataset.

Table S4 (separate file). Different expression genes in MCF7 cells overexpressing YAP1-S127A vs. control plasmid.

Table S5 (separate file). Annotation of TEAD4 ChIP sequence data in MCF7 cell line from ENCODE database (GSM1010860) via ChIP-Seek software.
